# Clinical and molecular findings in actin-related inborn errors of immunity: the middle East and North Africa registry

**DOI:** 10.3389/fgene.2025.1584681

**Published:** 2025-08-08

**Authors:** Zahra Chavoshzadeh, Shahrzad Fallah, Vahide Zeinali, Samin Sharafian, Samaneh Delavari, Mehrnaz Mesdaghi, Reda Djidjik, Brahim Belaid, Aydan Ikinciogullari, Sule Haskologlu, Figen Dogu, Ferah Genel, Nesrin Gulez, Safa Baris, Ahmet Ozen, Elif Karakoc-Aydiner, Ayça Kiykim, Zeynep Meric, Necil Kutukculer, Ayse Aygun, Guzide Aksu, Neslihan Edeer Karaca, Mehmet Geyik, Sevgi Keles, Ismail Reisli, Sukru Nail Guner, Rachida Boukari, Saliha Hakem, Reda Belbouab, Mohamed-Ridha Barbouche, Imen Ben-Mustapha, Najla Mekki, Meriem Ben-Ali, Ali Sobh, Marwa Elnagdy, Kamel Djenouhat, Azzeddine Tahiat, Hiba Mohammed Shendi, Amna Alkuwaiti, Gulnara Nasrullayeva, Tariq Alfars, Nashat Alsukaiti, Michel Massaad, Cybel Mehawej, Andre Megarbane, Carla Irani, Gehad Elghazali, Salem Al-Tamemi, Nisreen Khalifa, Raed Alzyoud, Sara Sebnem Kilic Gultekin, Hulya Kose, Hedieh Khodaverdy, Bibi Shahin Shamsian, Narges Eslami, Tooba Momen, Roya Sherkat, Soheila Aleyasin, Hossein Esmaeilzadeh, Hamid Ahanchian, Fereshte Salami, Saba Fekrvand, Loïc Dupre, Hans D. Ochs, Nima Rezaei, Waleed Al-Herz, Hassan Abolhassani

**Affiliations:** ^1^ Department of Allergy and Clinical Immunology, Mofid Children’s Hospital, Shahid Beheshti University of Medical Sciences, Tehran, Iran; ^2^ Research Institute for Children’s Health, Shahid Beheshti University of Medical Sciences, Tehran, Iran; ^3^ Research Center for Immunodeficiencies, Pediatrics Center of Excellence, Children’s Medical Center, Tehran University of Medical Sciences, Tehran, Iran; ^4^ Department of Medical Immunology, Beni Messous University Hospital Center, Faculty of Pharmacy, University of Health Sciences, Algiers, Algeria; ^5^ Department of Pediatric Immunology and Allergy, Ankara University School of Medicine, Ankara, Türkiye; ^6^ Department of Pediatric Immunology and Allergy, University of Health Sciences Dr. Behcet Uz Children’s Hospital, Izmir, Türkiye; ^7^ Division of Allergy and Immunology, Marmara University School of Medicine, Istanbul, Türkiye; ^8^ The Isil Berat Barlan Center for Translational Medicine, Marmara University School of Medicine, Istanbul, Türkiye; ^9^ Istanbul Jeffrey Modell Foundation Diagnostic Center for Primary Immune Deficiencies, Istanbul, Türkiye; ^10^ Department of Pediatric Allergy and Immunology, Faculty of Medicine, Istanbul University-Cerrahpasa, Istanbul, Türkiye; ^11^ Department of Pediatrics, Faculty of Medicine, Ege University, Izmir, Türkiye; ^12^ Division of Pediatric Allergy and Immunology, Meram Medical Faculty, Necmettin Erbakan University, Konya, Türkiye; ^13^ Department of Pediatrics, University Hospital of Mustapha Pacha, University of Algiers, Algiers, Algeria; ^14^ Department of Microbiology, Immunology and Infectious Diseases, College of Medicine and Health Sciences, Arabian Gulf University, Manama, Bahrain; ^15^ Laboratory of Transmission, Control and Immunobiology of Infections, Department of Immunology, Institut Pasteur de Tunis and University Tunis El Manar, Tunis, Tunisia; ^16^ Department of Pediatrics, Mansoura University Children’s Hospital, Faculty of Medicine, Mansoura University, Mansoura, Egypt; ^17^ Department of Biochemistry and Molecular Biology, Mansoura University Faculty of Medicine, Mansoura, Egypt; ^18^ Laboratory of Immunology, Department of Medical Biology, Rouiba Hospital, University of Algiers, Algiers, Algeria; ^19^ Department of Pediatrics, Tawam Hospital, Al-Ain, United Arab Emirates; ^20^ Department Immunology Research Laboratory, Azerbaijan Medical University, Baku, Azerbaijan; ^21^ Department of Immunology, Sheikh Khalifa Medical City, PureLab, PureHealth, and Faculty of Medicine, United Arab Emirates University, Al Ain, United Arab Emirates; ^22^ Department of Experimental Pathology, Immunology, and Microbiology, Faculty of Medicine, American University of Beirut, Beirut, Lebanon; ^23^ Division of Pediatric Infectious Diseases, Department of Pediatrics and Adolescent Medicine, American University of Beirut Medical Center, Beirut, Lebanon; ^24^ Department of Human Genetics, Gilbert and Rose-Marie Chagoury School of Medicine, Lebanese American University, Byblos, Lebanon; ^25^ Institut Jerome Lejeune, Paris, France; ^26^ Internal Medicine and Clinical Immunology, Hotel-Dieu de France Hospital, Saint Joseph University, Beirut, Lebanon; ^27^ Department of Child Health, Sultan Qaboos University Hospital, University Medical City, College of Medicine and Health Sciences, Sultan Qaboos University, Muscat, Oman; ^28^ NBK Children’s Hospital, Kuwait City, Kuwait; ^29^ Allergy, Immunology, and Rheumatology, Queen Rania Children’s Hospital, Amman, Jordan; ^30^ Division of Pediatric Allergy and Immunology, Department of Pediatrics, Faculty of Medicine, Uludağ University, Bursa, Türkiye; ^31^ Pediatric Congenital Hematologic Disorders Research Center, Research Institute for Children’s Health, Shahid Beheshti University of Medical Sciences, Tehran, Iran; ^32^ Department of Asthma, Allergy and Clinical Immunology, Child Growth and Development Research Center, Research Institute of Primordial Prevention of Non-Communicable Disease, Isfahan University of Medical Sciences, Isfahan, Iran; ^33^ Immunodeficiency Diseases Research Center, Alzahra University Hospital, Isfahan University of Medical Sciences, Isfahan, Iran; ^34^ Allergy Research Center, Shiraz University of Medical Sciences, Shiraz, Iran; ^35^ Allergy Research Center, Mashhad University of Medical Sciences, Mashhad, Iran; ^36^ Toulouse Institute for Infectious and Inflammatory Diseases (INFINITy), NSERM, CNRS, Toulouse University, Toulouse, France; ^37^ Department of Dermatology, Medical University of Vienna, Vienna, Austria; ^38^ Department of Pediatrics, Seattle Children’s Research Institute, University of Washington, Seattle, WA, United States; ^39^ Department of Pediatrics, College of Medicine, Kuwait University, Kuwait City, Kuwait; ^40^ Allergy and Clinical Immunology Unit, Pediatric Department, Al-Sabah Hospital, Kuwait City, Kuwait; ^41^ Division of Immunology, Department of Medical Biochemistry and Biophysics, Karolinska Institutet, Stockholm, Sweden

**Keywords:** actinopathies, thrombocytopenia, eosinophilia, inborn errors of immunity, primary immunodeficiency, genetic, immune dysregulation

## Abstract

**Background:**

The majority of monogenic inborn errors of immunity presenting as actinopathies were reported originally from the Middle East and North Africa (MENA) countries indicating a high prevalence of these entities in the region. However, their prognosis is unclear due to rarity and lack of comprehensive treatment outcomes.

**Methods:**

We evaluated clinical, immunological, and genetic abnormalities associated with 15 genetic entities of actinopathies. Based on the function of mutant genes in actin-regulatory pathways, patients were classified into CDC42- and RAC2-related subcategories.

**Results:**

A total of 503 individuals (29.5% females) from 17 countries were considered with a median age of 120 months. Although most patients presented initially with allergic phenotypes (37.7%), the most prevalent manifestations throughout the lifespan were infection in respiratory tracts (72.2%). Primary clinical diagnosis was mainly combined immunodeficiencies (48.3%) and the majority of cases were molecularly assigned to the CDC42 pathway (64.8%). The most common genetic defects were reported within the *DOCK8* (n = 209) followed by the *WAS* (n = 94) and the *CARMIL2* (n = 15) genes. Hematopoietic stem cell transplantation (HSCT) was conducted on 24.0% of patients, which significantly improved survival in patients with defects in *WAS, DOCK8* and *DOCK2*. Overall mortality was 23.0%, mainly due to sepsis and malignancy.

**Conclusion:**

Patients with defects in RAC2-associated regulators of actin usually present with late-onset symptoms due to normal immune profiles, but a higher rate of EBV and HPV infections, autoimmune cytopenia, asthma, and lymphoproliferation compared to defects in the CDC42 pathway. The severity of mutations in patients of the CDC42 group helps to estimate the prognosis of the disease and prioritization of HSCT.

## Introduction

Actin remodeling involves multiple proteins and contributes significantly to numerous cellular processes such as intracellular dynamic, cell migration, secretion of cellular production, endocytosis and rapid proliferation (SenGupta et al., 2021; Dominguez and Holmes, 2011). Defects in genes that encode actin and its pleiotropic regulatory proteins with restructuring activities in the cellular cytoskeleton, result in syndromic features known as “actinopathies” (Papa et al., 2020; Dupre and Prunier, 2023). As of today, more than 20 immune-related actinopathies have been characterized (Kamnev et al., 2021). The clinical manifestations are characteristics of a prominent immunodeficiency and immune dysregulation leading to the initial diagnosis of inborn error of immunity (IEI) in many of these syndromic patients (Dupre and Prunier, 2023; Kamnev et al., 2021). Examining these various genetic defects reveals the large efficiency of the molecular regulation of actin remodeling in diverse leukocyte subsets, in comparison to other types of cells and tissues (von der Ecken et al., 2016; Merino et al., 2020). Moreover, some of these actin regulators have immune-restricted expression. Both myeloid and lymphoid cells at the cellular scale are highly dependent on actin remodeling for the establishment of immunological synapses, surface receptor clustering and signaling, organelle shuffling, cell protrusion for antigen sensing, diapedesis and interstitial infiltration, pathogen engulfment, and secretion of effector or cytolytic molecules (Dominguez and Holmes, 2011; Burbage et al., 2015; Obino et al., 2016). Actin remodeling also controls cellular deformations and ensures cell integrity in the context of homing and positioning of immune cells from the bone-marrow to peripheral tissues and secondary lymphoid organs (Merino et al., 2020; Kunimura et al., 2020).

Upon activation of leukocytes via surface antigen receptors or integrins, actin polymerization can be triggered from actin monomers by the action of two nucleation factors known as formins and actin-related protein 2/3 (ARP2/3) which initiates the branching daughter filaments on the existing actin filament (Merino et al., 2020). Within the cortex, the area underneath the plasma membrane of leukocytes, receptor signaling is delivered by guanosine triphosphatases/GTPases (including CDC42, RAC2, RHOG, RHOH) (Dupre and Prunier, 2023; Obino et al., 2016). The GTPases, particularly CDC42 and RAC2, are orchestrated by distinct associated regulators (including DOCK2, DOCK8, RASGRP1, VAV, NCK, NIK). Defects in both CDC42 and RAC2 GTPases have been linked with actinopathies but associated with distinct immune disorders. CDC42 deficiency usually presents with multisystemic autoinflammation which may progress to myelofibrosis/proliferation, hemophagocytic lymphohistiocytosis and enterocolitis (Takenouchi et al., 2015; Takenouchi et al., 2016). In contrast, RAC2 deficiency usually causes T cell dysfunction, myeloid abnormalities and leukocyte adhesion defects (Donkó et al., 2024; Alkhairy et al., 2015). This observation indicates an unique conspicuous downstream signaling and immune dependency to each of these two GTPases. Physiologically, these GTPases activate actin-binding proteins or subunits of actin-binding protein complexes (including WASP, WIP, HEM1, ARPC1B, CORO1A, WDR1, MSN, DIAPH1, MKL1, MYH9) and direct regulators of actin-binding proteins (including PSTPIP1, CARMIL2, STK4). These proteins are involved in ARP2/3-dependent actin filament branching (WASP, WIP, PSTPIP1, HEM1 and ARPC1B), actin turnover (CORO1A, WDR1), actin cross-linking with the plasma membrane (MSN), actin elongation (DIAPH1) and actin capping (CARMIL2) (Papa et al., 2020; Burbage et al., 2015; Kunimura et al., 2020; Ambruso et al., 2000; Janssen et al., 2016). Lymphocytes, neutrophils, monocytes/macrophages and dendritic cells rely on this critical cytoskeleton remodeling system. However, their regulation by upstream pathways is cell-type specific and is responsible for the specialized functions of these cells (Kamnev et al., 2021). Therefore, defects in these proteins may present with combined immunodeficiencies (T cell defects), phagocytosis defects (neutrophil and macrophage defects), immune dysregulation of adaptive immunity, and autoinflammation caused by an imbalance of innate immunity. However, based on the classification issued by the International Union of Immunological Societies (IUIS), most actinopathies are classified as syndromic combined immunodeficiencies (Dupre and Prunier, 2023; Tangye et al., 2022).

Actin dysregulation has been linked to an increasing number of genes by using next-generation sequencing on unsolved IEI patients. While Wiskott-Aldrich syndrome (WAS) and a few common monogenic actinopathies have been clinically and immunologically well explored, there is limited information available for other actinopathies because of their rarity (Dupre and Prunier, 2023). So far, each entity has been studied separately. Gathering clinical and biological parameters, as well as treatment experience, in a systematic manner across these entities would allow a better understanding of their shared and distinctive traits, ultimately allowing for accelerated diagnosis and implementation of more tailored treatments. Most of these disorders are inherited with autosomal recessive patterns and therefore are more common in regions where consanguineous marriages are prevalent such as the Middle East and North Africa (MENA). In this collaborative study, we assembled a unique patient cohort from the MENA region covering 15 actinopathies and aimed to report data pertaining to clinical monitoring, laboratory findings and treatment responses.

## Materials and methods

### Study design

This survey was designed to recruit patients with actin-related IEI cared for in MENA countries. This retrospective, longitudinal, multicenter investigation was conducted between February 2023 and July 2023 and included countries that participated in the MENA-IEI registry (Aghamohammadi et al., 2021; Baris et al., 2023). Patient data were analyzed without restrictions as to the initial diagnosis and included all IUIS categories (Tangye et al., 2022). Following the establishment of a research agreement with centers in MENA countries, investigators from the region were invited to enroll their patients. The study was approved by the ethics committee of Tehran University of Medical Sciences, Tehran, Iran, following the guidelines of the Helsinki Declaration. Patients or their parents provided informed consent according to the participating centers’ local institutional review board (IRB).

### Study population

**FIGURE 1:**
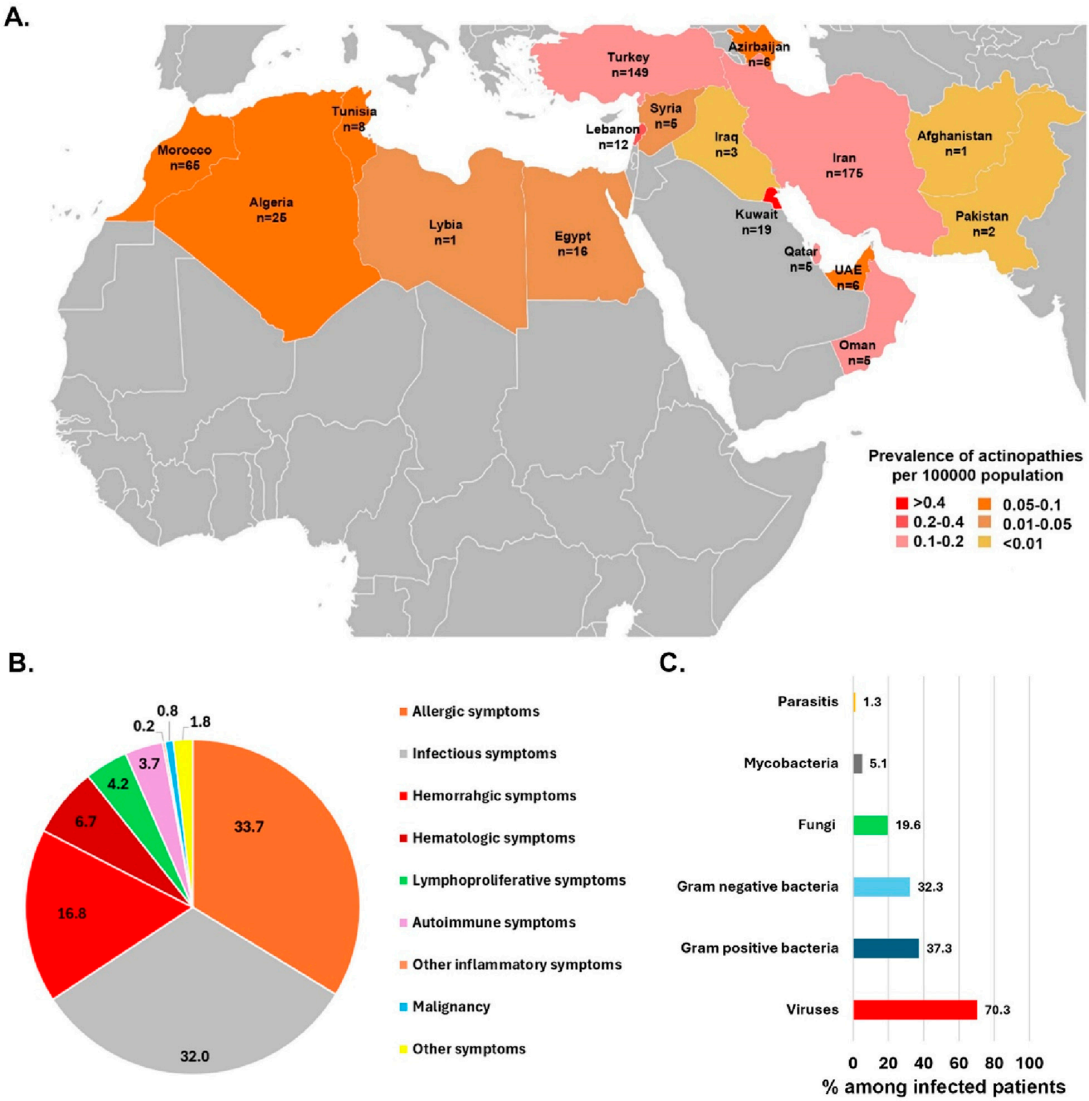
**(A)** An illustration of countries from the MENA region and the prevalence and number of actin-related inborn errors of immunity cases contributed to the study originated from 17 countries. **(B)** The frequency of first presentations in actin-related inborn errors of immunity patients. **(C)** Percentage of different pathogens isolated from infected patients with actinopathies.

All patients were clinically diagnosed with IEI who had characteristic findings of immune-related actinopathies, based on the MENA diagnostic guideline (Baris et al., 2023) and the IUIS classification (Tangye et al., 2022). The inclusion criteria were: (i) Established clinical diagnosis of IEI with cytoskeletal abnormalities based on the medical presentation, laboratory data, imaging and pathologies (Bousfiha et al., 2020; Tangye et al., 2021); AND EITHER (ii) Presence of a confirmed pathogenic variants in one of the genes implicated in cytoskeletal abnormalities and actinopathies; including WASP deficiency (*WAS* mutations) WIP deficiency (*WIPF1* mutations)*,/*ARP2-3 deficiency (*ARPC1B* mutations)*,/*STK4 deficiency (*MST1* mutations)*,* CORONIN1A deficiency (*CORO1A* mutations)*,* CARMIL2 deficiency (*RLTPR* mutations)*,* HEM1 deficiency (*NCKAP1L* mutations)*,* as well as, DOCK8, DOCK2, RASGRP1, RHOH, RHOG, CDC42, PSTPIP1, CEBPE, MSN, ACTB, WDR1, RAC2 and MKL1 deficiencies based on American College of Medical Genetics and Genomics (ACMG) criteria (Richards et al., 2015); OR (iii) Genetically unsolved patients with classical presentation of WAS (eczema, recurrent bacterial or viral infections, autoimmunity, malignancy, specific antibody defects, positive family history of WAS and male patient with thrombocytopenia less than 100,000/ul and small platelets volume <7.5 fL); AND (iv) Patients originating from one of the countries in the MENA region (Figure 1A) (Baris et al., 2023). The exclusion criteria were: (i) Significant incomplete documentation of demographic, clinical, or immunological features; OR (ii) Digenic or oligogenic defects.

For genetic evaluation of patients, genomic DNA was isolated from peripheral blood following established protocols in each center and followed the consensus MENA diagnosis and management guidelines for Inborn Errors of Immunity (Baris et al., 2023). Briefly, for patients displaying classic clinical features indicative of a particular WASP deficiency, Sanger sequencing was used to analyze the 12 exons of the candidate gene as published previously (Derry et al., 1995). In cases where Sanger sequencing did not yield a diagnosis, or when clinical features overlapped with multiple genetic conditions, whole exome sequencing (WES) was conducted with no restriction regarding capturing kits (e.g., Agilent SureSelect V4-V8, NimbleGen SeqCap V3, IDT xGEN Exome Research Panel v1.0, and Roche Prime Exome) and sequencing platforms (e.g., Beijing Genomics Institute BGISEQ-500, Illumina HiSeq, NovaSeq 6000, NovaSeq X, and NextSeq 2000 Systems) at it varies within countries and region based on the available resources, year of diagnosis and local policies and agreements. Harmonized analysis of WES data was performed using a previously described pipeline, with batch effect correction and normalization and including an average on-target read depth of 50× including the detection of larger deletions, using normalized mean exon coverage (Abolhassani et al., 2018).

### Data collection

An encrypted spreadsheet form was used to collect epidemiological, clinical and laboratory information of patients including demographic data (sex, ethnicity, consanguinity, age at onset, age at diagnosis, year of birth, age at diagnosis, delay of diagnosis, age at last visit, family history, and live/death status), and clinical information (initial symptoms, clinical diagnosis, type of infections, autoimmune and autoinflammatory manifestations, atopic presentations, hematologic/immunologic features, malignancy type and age of onset of each of these phenotypes). Details of treatment modalities were obtained, including immunoglobulin (Ig) therapy, antibiotic prophylaxis, biologic or immunosuppressive drugs, hematopoietic stem cell transplantation (HSCT) and type of transplant. Not all the detailed items were available for every patient. Laboratory data were compiled for complete blood count (CBC), mean platelet volume (MPV), immune subsets and serum Ig levels. Genetic studies focused on the type of mutations, zygosity and identification of hot spot variants typical for the MENA region, as described previously (Jamee et al., 2022). Mutations due to nonsense or frameshift variants were defined as severe, while in-frame deletion/insertion, splicing, or missense mutations were classified as milder variants.

### Statistical analysis

**FIGURE 2:**
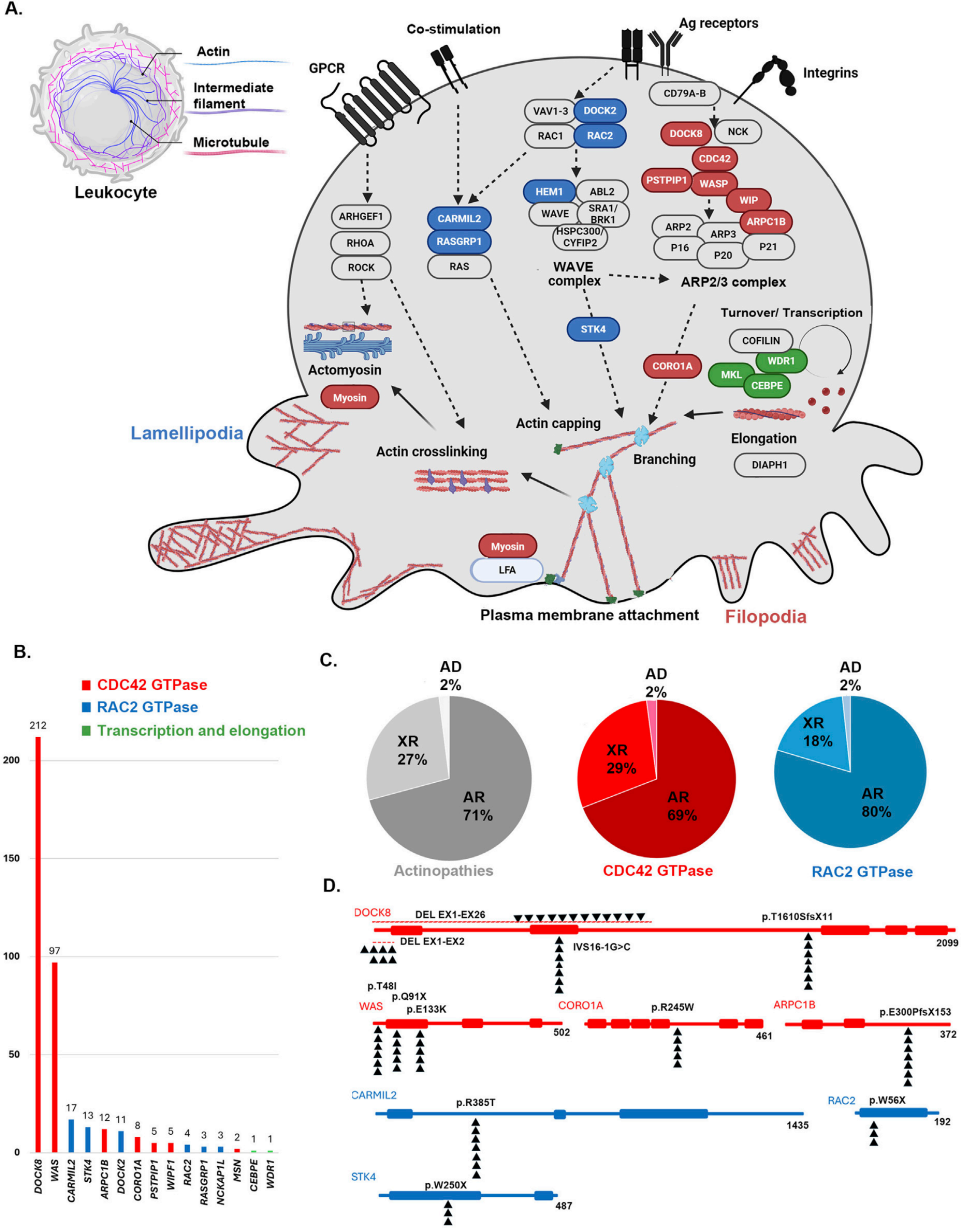
**(A)** Schematic pathway of actin-remodeling in the leukocyte via two main pathways. An opportunity for fresh actin polymerization to generate membrane protrusions at the leading edge is created by the collapse of the cortical actin network to the side of cells occupied by microtubule organizing centers by two main pathways associated with The Rho family of tiny G-proteins, which includes RAC2 (blue colors) and Cell division control protein 42 homolog (CDC42, red colors). Activated CDC42 pathway generates filopodia by inducing the WAVE complex and the RAC2 pathway promotes lamellipodia via ARP2/3 complex. Few genes also are known with an important role in the recycling and transcription of monomers of actins (green colors) **(B)** The frequency of genes, **(C)** inheritance pattern (AR: autosomal recessive, XR: X-lined recessive, AD: autosomal dominant) and **(D)** the most frequent variants associated with monogenic actin-related inborn errors of immunity mainly associated with defects in RAC2 and CDC42 GTPase pathways.

Data from each center was assessed by one investigator who evaluated each of the completed questionnaires for ambiguities and compliance with inclusion criteria, contacting the respective center for clarification. Final data were transferred to SPSS (v. 26.0, Chicago, IL) and R statistical systems (version 3.4.1., R Foundation for Statistical Computing, Vienna, Austria). Patients were subdivided into different subcategories based on the function of the mutated genes affecting actin cytoskeleton pathways involving the most important GTPases (CDC42 and RAC2, Figure 2A) as suggested previously based on animal-models and *in-vitro* studies (Papa et al., 2020). Descriptive statistical analyses were performed for quantitative and qualitative variables. Comparisons between groups were determined using standard statistical tests (Chi-square, Fischer’s exact test, Mann-Whitney and Kruskal–Wallis). The Kaplan-Meier survival estimator was used to assess overall survival or to compare mortality rates among different groups of actinopathies. A *p*-value of less than 0.05 was considered statistically significant.

## Results

### Clinical outcome, immunologic profile, and genetic analysis of the actinopathy cohort

The study group included 503 patients (29.5% females) from 17 countries across the MENA region. These patients represented 15 actin-related IEI entities, which were classified as related either to the CDC42 or the RAC2 pathway (Table 1, see also Figure 2A). The first part of the analysis globally considers all patients, whereas the second part of the analysis focuses on the specificities of the patients belonging to the CDC42 pathway and RAC2 pathway subgroups. Ethnicity was Persian in 34.7% of enrolled subjects followed by Turkish (29.6%) and Maghrebian Arab (12.9%, Figure 1A). Consanguineous marriages were identified in the parents of 59.2% of our cohort, and a positive family history of IEI was documented in 61.5%. The median (interquartile range IQR) age at symptom onset was 4 (0.1–270) months and most patients (79.9%) presented during infancy. The most common initial presenting symptoms were allergic manifestations (33.7%), followed by infections (32.0%), and hemorrhagic manifestation (16.8%, Figure 1B). Eczema/atopic dermatitis was recognized as the most prevalent primary symptom (30.5%) seen in this actinopathy cohort. Among the infectious manifestations, lower respiratory tract infections were most prevalent (lower RTI 23.9%), followed by recurrent upper RTI involvement (13.3%). The median age at diagnosis was 36 (IQR 0.2–300) months. Of note, patients with actinopathies in the MENA region reported a median diagnostic delay of 19 months. Only 3 patients were diagnosed in adulthood. At the time of the study, most patients were in middle childhood and 23.0% were deceased due to complications related to actinopathies (Table 1; Supplementary Table S1).

**TABLE 1:** Demographic data in 503 patients with actin-related inborn errors of immunity.

Parameters	Total (n = 503)	Unsolved (n = 109)	Other actinopathies (n = 2)	CDC42 pathway (n = 341)	RAC2 pathway (n = 51)	*P*-value*
Sex, M/F	355/148	109/0	1/1	221/120	24/27	0.014*
Ethnicity						<0.001*
Persian (%)	175 (34.7)	35 (32.1)	0 (0)	118 (34.6)	22 (43.1)	
Turkish (%)	149 (29.6)	5 (4.8)	1 (50)	127 (37.2)	16 (31.3)	
Maghrebi Arab (%)	65 (12.9)	50 (45.8)	0 (0)	9 (2.6)	6 (11.7)	
Algerian Arab (%)	25 (4.9)	14 (12.8)	0 (0)	9 (2.6)	2 (3.9)	
Kuwaiti Arab (%)	19 (3.7)	2 (1.8)	0 (0)	17 (4.9)	0 (0)	
Egyptian Arab (%)	16 (3.1)	1 (0.9)	0 (0)	14 (4.1)	1 (1.9)	
UAE Arab (%)	6 (1.2)	0 (0)	0 (0)	6 (1.7)	0 (0)	
Lebanese (%)	12 (2.3)	1 (0.9)	0 (0)	11 (3.2)	0 (0)	
Tunisian Arab (%)	8 (1.6)	0 (0)	0 (0)	8 (2.3)	0 (0)	
Azeri (%)	6 (1.0)	0 (0)	0 (0)	5 (1.4)	1 (1.9)	
Omani Arab (%)	5 (1.0)	0 (0)	0 (0)	2 (0.5)	3 (5.8)	
Qatari Arab (%)	5 (1.0)	0 (0)	1 (50)	4 (1.1)	0 (0)	
Syrian Arab (%)	5 (1.0)	0 (0)	0 (0)	4 (1.1)	1 (1.9)	
Iraqi Arab (%)	3 (0.6)	0 (0)	0 (0)	3 (0.8)	0 (0)	
Pakistani (%)	2 (0.4)	0 (0)	0 (0)	2 (0.5)	0 (0)	
Lybian Arab (%)	1 (0.2)	1 (0.9)	0 (0)	0 (0)	0 (0)	
Afghani (%)	1 (0.2)	0 (0)	0 (0)	1 (0.3)	0 (0)	
Age at study, month, median (IQR)	120 (0.1–444)	55 (0.1–217)	216	107.5 (0.1–372)	147 (4–295)	0.03*
Age at onset, month, median (IQR)	4.0 (0.1–270)	2.5 (0–170)	2 (1–3)	4.5 (0–270)	29 (1–216)	<0.001*
Age at diagnosis, month, median (IQR)	36 (0.2–300)	11 (0–216)	105	36 (0.1–300)	132 (5–264)	<0.001*
Diagnosis delay, month, median (IQR)	19 (0.1–264)	8 (0–214)	102	25.5 (0.1–264)	97 (0.1–207)	<0.001*
Age at first infection, month, median (IQR)	6 (0.1–276)	6 (2–276)	-	6 (0.1–192)	30 (3–120)	0.03*
Age at first autoimmunity, month, median (IQR)	24 (0.1–340)	11.5 (1–56)	-	21 (0–187)	51 (2–340)	0.011*
Age of first lymphoproliferation, month, median (IQR)	38 (2–228)	5 (2–176)	-	70 (2–144)	132 (48–228)	<0.01*
Age of first malignancy, month, median (IQR)	95 (33–176)	158.5 (141–176)	-	92.5 (33–156)	87 (36–96)	0.12
Age of onset of eczema, month, median (IQR)	4 (0.1–276)	3 (0.1–276)	-	6 (1–144)	31 (3–168)	0.024*
Age of first allergic disorder except eczema, month, median (IQR)	10 (1–168)	6 (6–6)	-	8 (1–96)	36 (4–168)	0.008*
Mortality (%)	116 (23.0)	46 (42.2)	0 (0)	67 (19.6)	3 (5.8)	0.016*

M, male; F, female; D, dead; A, alive; IQR, interquartile range. * All ages are in month. * A comparison between CDC42 and RAC2 groups performed and p-value<0.05 is considered as significant. UAE: United Arab Emirates.

Notably, infections (90.1%) were the predominant manifestations observed in patients throughout their lifespan, succeeded by allergic (72.0%) and hematologic (52.2%) disorders (Table 2). Infections predominantly affected the respiratory tracts (72.2%), followed by skin (48.1%) and gastrointestinal tracts (26.4%). Details of infectious complications were available for 340 patients (Supplementary Tables S2–S6). Pneumonia was the most common lower RTI, affecting 44.7%, and leading to bronchiectasis in 12.1% of patients with actinopathies (Supplementary Table S2). Dermatologic infections were mostly due to viral agents (25.7%, mainly *Molluscum contagiosum, Herpes simplex virus* and *Human papillomavirus*) [HPV] and to a lesser extent due to fungal (13.8%, mainly *Candida albicans*) and bacterial agents (9.1%, mainly presented with skin abscesses Supplementary Table S3). *Streptococcus pneumoniae* and *Staphylococcus aureus* were the predominant Gram-positive bacteria. Mycobacteria were detected in 5.1% of the cases, predominantly represented by *Mycobacterium bovis* in patients with BCG vaccine side effects (2.4%).

**TABLE 2:** Clinical manifestations of 503 patients during the course of actin-related inborn errors of immunity.

Clinical presentations	Total (n = 503)	Unsolved (n = 109)	Other actinopathies (n = 2)	CDC42 pathway (n = 341)	RAC2 pathway (n = 51)	*P*-value
Infections presentations, n (%)	453 (90.1)	99 (90.8)	2 (100)	314 (92.0)	38 (74.5)	0.28
Respiratory infections, n (%)	363 (72.2)	73 (67)	1 (50)	258 (75.6)	31 (60.7)	0.43
Recurrent upper respiratory infections, n (%)	256 (50.9)	49 (45)	1 (50)	184 (53.9)	22 (43.1)	0.6
Lower respiratory infections, n (%)	225 (44.7)	32 (29.4)	1 (50)	175 (51.3)	17 (33.3)	0.11
Dermatologic infections, n (%)	242 (48.1)	35 (32.1)	2 (100)	185 (54.2)	20 (39.2)	0.26
Gastrointestinal infections, n (%)	133 (26.4)	46 (42.2)	1 (50)	77 (22.5)	9 (17.6)	0.73
Genitourinary infections, n (%)	27 (5.4)	8 (7.3)	1 (50)	18 (5.2)	0 (0)	0.24
Nervous system infections, n (%)	24 (4.8)	4 (3.7)	0 (0)	18 (5.2)	2 (3.9)	0.82
Ophthalmic infections, n (%)	15 (3.0)	0 (0)	1 (50)	11 (3.2)	3 (5.8)	0.23
Musculoskeletal infections, n (%)	13 (2.6)	3 (2.8)	0 (0)	10 (2.9)	0 (0)	0.61
Systemic infections, n (%)	86 (17.1)	14 (12.8)	0 (0)	69 (20.2)	3 (5.8)	0.03^a^
Autoimmune presentations, n (%)	134 (26.6)	37 (33.9)	0 (0)	87 (25.5)	10 (19.5)	0.67
Allergic and atopic presentations, n (%)	362 (72.0)	72 (66.1)	0 (0)	263 (77.1)	27 (52.9)	0.02^a^
Lymphoproliferative presentations, n (%)	96 (19.1)	24 (22)	0 (0)	55 (16.1)	17 (33.3)	0.001^a^
Hematological presentations, n (%)	263 (52.2)	93 (85.3)	1 (50)	151 (44.2)	18 (35.2)	0.65
Malignant/oncologic presentations, n (%)	30 (6.0)	2 (1.8)	0 (0)	24 (7.0)	4 (7.8)	0.62

^a^ A comparison between CDC42 and RAC2 groups performed and p-value<0.05 is considered as significant.

Eczema took precedence as the most frequent allergic presentation (67.9%), pursued by food allergies (15.6%) and asthma (15.6%). Hemorrhagic manifestations were observed in 17.5% of patients, whereas cytopenia was reported in 42.7% of cases (Supplementary Table S5). Thrombocytopenia (33.5%) emerged as the most frequent type of cytopenia. Of note, 26.7% of patients exhibited autoimmune manifestations, indicating that the underlying pathogenesis of cytopenia was due to autoimmunity in only 6.5% (Supplementary Table S6). At least one lymphoproliferative manifestation was observed in 19.1% of patients and lymphadenopathy (11.2%) was the primary one. Malignancy was observed in 5.9% of individuals, with lymphoma being the predominant type of malignancy (2.6%, Table 1; Supplementary Table S5). The median age at onset of eczema (4 [0–276] months) was found to be lower compared to other manifestations followed by the infectious presentation at 6 (2–276) months. The highest median age of disease occurrence was found in patients with malignancy, 95 (33–176) months (Supplementary Figure S1).

Among our group of 503 patients, the main IUIS categories at the time of diagnosis were combined immunodeficiencies (48.3%), syndromic combined immunodeficiencies (45.1%), and immune dysregulation (3.9%). Phagocytosis defects and autoinflammation were assigned as the first clinical diagnoses of only 8 and 5 patients, respectively. Table 3 summarizes the immunological evaluation at the time of diagnosis before initiation of treatment in patients with actinopathies. Lymphopenia was documented in 23.7% of patients (mean of lymphocyte count: 3,689.3 ± 912.5/μL), neutropenia in 6.1% (mean of neutrophil count: 5,551.4 ± 3,315.6/μL), anemia in 63.4% (mean hemoglobin: 12.0 ± 4.1 g/dL), low mean platelet volume in 38.5% (mean MPV:7.7 ± 1.80 fL). Lymphocyte subset analysis indicated low CD3^+^ T cells in 50.4% (mean: 2,107.4 ± 1,652.1/μL), low CD4^+^ T cells in 61.5% (mean:1,129.0 ± 1,056.1/μL), low CD8^+^ T cells in 40.7% (mean: 894.1 ± 414.3/μL), low Tregs in 80% (mean % of Treg cell:1.47 ± 1.02), low CD19^+^ B cells in 33.3% (mean: 1,399.0 ± 717.2/μL), low NK cells in 14.8% (mean: 501.0 ± 275.7/μL) and high CD21^low^ B cells in 48.4% (mean % of CD21^low^ B cell: 3.72 ± 2.4). Analysis of Ig levels also revealed low IgG in 11.2% (mean: 2,607.8 ± 1,573.5 mg/dL), low IgA in 8.8% (mean: 327.5 ± 163.2 mg/dL), low IgM in 44.1% (mean: 79.9 ± 29.4 mg/dL) and high IgE levels in 73.2% (mean: 2,615.9 ± 1,464.8 IU/mL, Table 3).

**TABLE 3:** Immunologic profile in 503 patients during the course of actin-related inborn errors of immunity.

Parameters	Total (n = 503)	Unsolved (n = 109)	Other actinopathies (n = 2)	CDC42 pathway (n = 341)	RAC2 pathway (n = 51)	*P*-value*
Absolute leukocytes, cell/µL, median (SD)	11,696.6 (6390.5)	10,993.6 (5,306.2)	6000	12,615.4 (6886.1)	8,131.9 (4,280.2)	<0.001*
Absolute lymphocytes, cell/µL, median (SD)	3,689.3 (2,912.5)	4,367.2 (3,887.2)	2000	3,485.1 (2,435.4)	3,133.1 (2,207.4)	0.44
Absolute neutrophil count, cells/µL, median (SD)	5,551.4 (3,315.6)	5,343.3 (2,841.9)	2,200	5,914.4 (3,565.6)	4,014.3 (2,277.8)	0.003*
Absolute eosinophil count, cells/µL, median (SD)	850 (320–2,600)	520 (220–1,050)	117	2,600 (735–5,120)	172 (23–258)	<0.001*
Platelets, cells/µL, median (SD)	217,422.3 (215,193.3)	47,323.9 (61,176)	432,000	262,020.6 (230,486.5)	324,433.3 (104,430.4)	0.14
Mean platelet volume, fl (SD)	7.7 (1.8)	6.9 (1.4)	8.6	8.2 (1.9)	8.6 (1.3)	0.47
Hemoglobin, g/dL, median (SD)	12 (4.1)	10.1 (2.2)	13	12.3 (4.9)	15.1 (5.3)	0.33
CD3^+^ T cells, % of lymph, median (SD)	37.6 (30.2)	13.4 (26.3)	78	44.9 (25.8)	46.4 (36.2)	0.78
Absolute T cell count, cells/µL, median (SD)	2,107.4 (1,652.1)	2,572 (1,619.2)	1,560	1924.8 (1,625.2)	2,180.1 (1751.8)	0.42
CD4^+^ T cells, % of lymph, median (SD)	26.0 (47.7)	12.7 (4.1)	42	27.1 (18.2)	48.6 (12.9)	0.02*
Absolute T helper count, cells/µL, median (SD)	1,129.0 (1,056.1)	1,343.8 (970.4)	840	1,043.3 (510.4)	1,176 (1,082.7)	0.63
CD8^+^ T cells, % of lymph, median (SD)	21.3 (10.5)	15.6 (13.5)	35	22.3 (18.3)	50 (26.3)	0.001*
Absolute T cytotoxic, cells/µL, median (SD)	894.1 (414.3)	911.4 (366)	700	894 (389.9)	862.6 (612.1)	0.87
CD19^+^ B cells, % of lymph, median (SD)	16 (11.5)	15.3 (12.1)	15	20.8 (18)	14.7 (11.3)	0.04*
Absolute B cell count, cells/µL, median (SD)	1,399.0 (717.2)	1,663.7 (1,358.3)	300	1,426.3 (911.6)	748.8 (589.3)	0.02*
NK cell, % of lymph, median (SD)	9.3 (11.2)	1.9 (5.9)	4	11.9 (9.4)	8.1 (7.9)	0.09
Absolute NK cell count, cells/µL, median (SD)	501.0 (275.7)	814.8 (433.5)	136	432.3 (156)	304.2 (237.7)	0.13
CD4^+^CD25+FOXP3+ cells, % of CD4^+^ T cells, median (SD)	1.47 (1.02)	2.3 (1.1)	2.5	2.82 (1.92)	0.90 (0.76)	0.08
CD21^low^ B cell, % of B cells, median (SD)	3.72 (2.44)	3.95 (1.61)	NI	6.03 (4.69)	2.85 (1.06)	0.07
IgG, mg/dL, median (SD)	2,607.8 (1,573.5)	5,513.1 (2,790.1)	2,520	1777.7 (938.8)	989.3 (476.1)	0.63
IgA, mg/dL, median (SD)	327.5 (163.2)	534.7 (298.1)	484	267.1 (110.9)	225.1 (206)	0.79
IgM, mg/dL, median (SD)	79.9 (29.4)	77.9 (67.4)	222	75.5 (48.5)	108.2 (91.1)	0.23
IgE, IU/mL, median (SD)	2,615.9 (1,464.8)	695.5 (593.9)	110	3,414.3 (1,227.8)	213.1 (100.3)	0.01*

Lymph; lymphocyte, SD; standard deviation, NI: Not indicated. * A comparison between CDC42 and RAC2 groups performed and p-value<0.05 is considered as significant.

**FIGURE 3:**
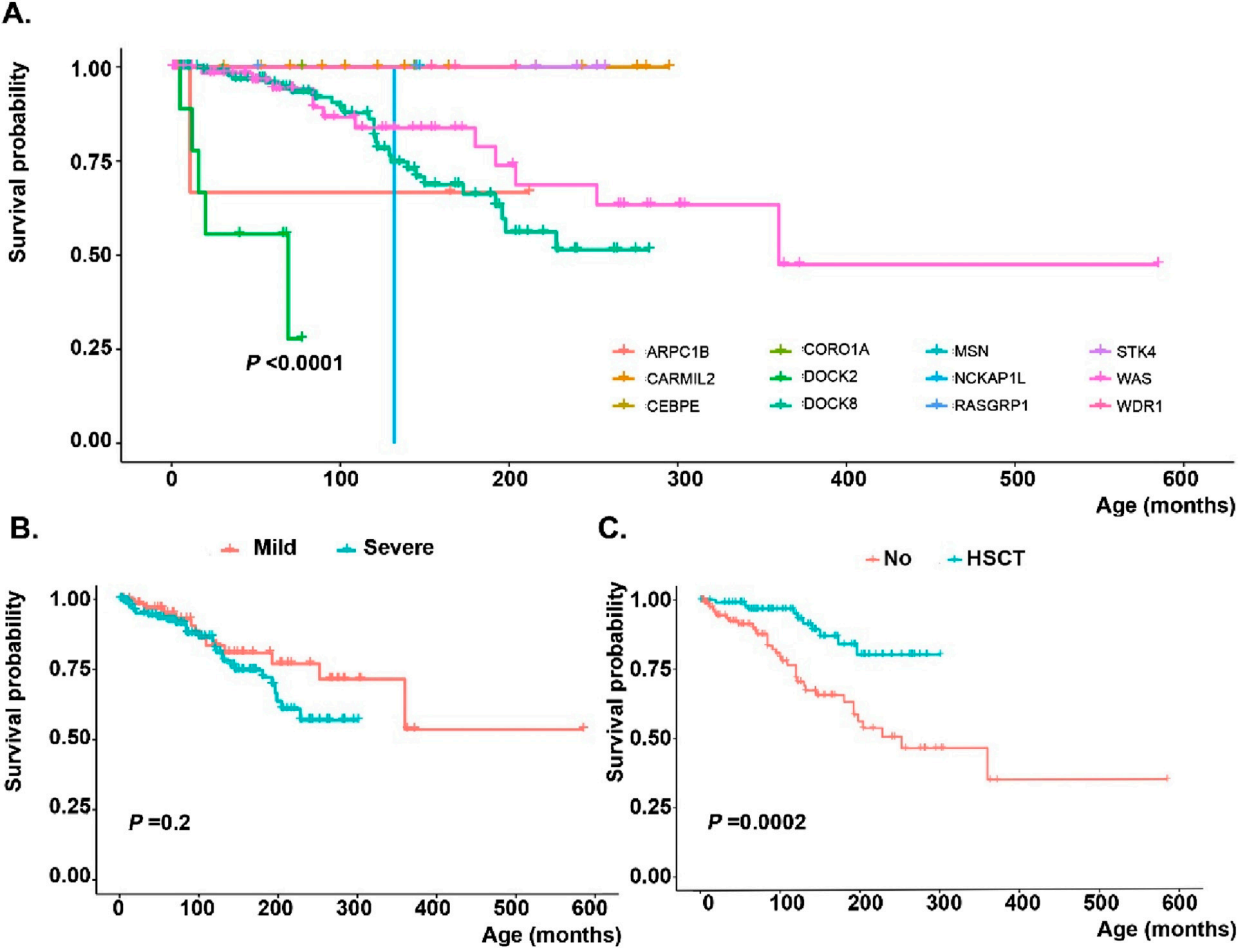
Survival analysis of monogenic actin-related inborn errors of immunity patients in the MENA region based on **(A)** genetic defects, **(B)** severity of mutations (nonsense or frameshift variants assigned as severe, while in-frame deletion/insertion, splicing, or missense mutations depicted as mild) and **(C)** hematopoietic stem cell transplantation.

Most of the patients (89.4%) received intravenous Ig therapy, while a smaller proportion (25.1%) required systemic corticosteroid treatment. Most patients (93.0%) received antibiotic prophylaxis, and 121 patients (24.0%) had undergone HSCT (Table 4). The main countries with frequent HSCT-reported actinopathies were Turkey (n = 75), Iran (n = 12), Algeria (n = 11) and Kuwait (n = 10) in the MENA region. The median age at transplantation and follow-up time after transplantation until data abstraction were 69 (3–303) and 55 (1–254) months, respectively. Most donors were HLA-matched and unrelated (48.2%), followed by HLA-matched siblings (28.2%), matched family members (12.9%), and haploidentical donors (10.6%). Bone marrow was the predominant graft source (79.2%), followed by peripheral blood (19.4%) and cord blood (1.4%). Among transplanted actinopathy patients, almost all patients underwent a conditioning regimen and 51.2% received graft versus host disease prophylaxis. The most commonly used conditioning regimen consisted of Fludarabine combined with Busulfan 17.4%, followed by Treosulfan in conjunction with Fludarabine 11.6%. The majority of transplanted individuals are presently in good post-transplantation conditions. At the same time, 4 patients encountered graft-rejection, and 7 patients died of HSCT-related complications, indicating a highly significant impact of HSCT on the survival rate of patients with actinopathies (*p* = 0.0002, Figure 3). The main cause of death in non-HSCT deceased patients was sepsis (25%), while malignancy ranked second (6.7%).

**TABLE 4:** Treatment modality data in 503 patients during the course of actin-related inborn errors of immunity.

Parameters	Total (n = 503)	Unsolved (n = 109)	Other actinopathies (n = 2)	CDC42 pathway (n = 341)	RAC2 pathway (n = 51)	P-value
IG replacement therapy (%)	450 (89.4)	107 (98.1)	2 (100)	301 (88.2)	40 (78.4)	0.091
Corticosteroids treatment (%)	126 (25.1)	4 (3.6)	0 (0)	112 (32.8)	10 (19.6)	<0.001^a^
Antibiotic prophylaxis (%)	468 (93.0)	104 (95.4)	0 (0)	318 (93.2)	46 (90.1)	<0.001^a^
Biologic or Immunosuppressive (%)	121 (24.0)	16 (14.6)	2 (100)	92 (26.9)	11 (21.5)	0.017^a^
HSCT (%)	121 (24.0)	13 (11.9)	0 (0)	103 (30.2)	5 (9.8)	<0.001^a^
HSCT source						
Bone marrow (%)	57/72 (79.2)	1/2 (50)	-	51/65 (78.5)	5/5 (100)	0.637
Peripheral blood (%)	14/72 (19.4)	1/2 (50)	-	13/65 (20)	0 (0)	
Umbilical cord (%)	1/72 (1.4)	0/2 (0)	-	1/65 (1.5)	0 (0)	
HSCT type						
Matched unrelated donor (%)	41/85 (48.2)	2/3 (66.7)	-	35/77 (45.5)	4/5 (80)	0.729
Matched sibling donor (%)	24/85 (28.2)	1/3 (4.2)	-	22/77 (28.6)	1/5 (4.2)	
Matched family donor (%)	11/85 (12.9)	0 (0)	-	11/77 (14.3)	0 (0)	
Haploidentical (%)	9/85 (10.6)	0 (0)	-	9/77 (11.7)	0 (0)	
HSCT HLA matching						
Partial (%)	17/86 (19.8)	0 (0)	-	16/78 (20.5)	1/5 (20)	0.839
Full (%)	64/86 (74.4)	3/3 (100)	-	57/78 (73.1)	4/5 (80)	
Haploidentical (%)	9/85 (10.4)	0 (0)	-	9/78 (11.5)	0 (0)	
GVHD prophylaxis (%)	62/121 (51.2)	2/12 (16.7)	-	55/104 (52.9)	5/5 (100)	0.005^a^

^a^ A comparison between CDC42 and RAC2 groups performed and p-value<0.05 is considered as significant.

HLA, Human leukocyte antigens; HSCT, Hematopoietic stem cell transplantation; GVHD, Graft-versus-host disease.

A molecular diagnosis could not be established in 109 participants. Most of them were of Maghrebian Arab ethnicity (45.8%), followed by Persian ethnicity (32.1%) and Algerian ethnicity (12.8%). The median age at onset of symptoms and at diagnosis were 2.50 (0–170) months and 11 (0–216) months, respectively, suggesting a more severe phenotype in this molecularly undefined group of patients as most had developed complications during infancy (Table 1; Supplementary Table S1). This group of patients with actinopathies presented a higher rate of gastrointestinal and genitourinary infections and hematologic and autoimmune presentations when compared with the remaining cohort of cases with defined molecular defects (Table 2). Similarly, the overall survival rate of this group was 57.8%, indicating that early mortality observed in this group hindered the genetic diagnosis, due to the absence of biobanking in specific countries for post-mortem sequencing.

Pathogenic or likely pathogenic variants based on ACMG criteria were detected in 394 individuals affecting 15 distinct genes; of which *DOCK8* (n = 212, 53.8%) and *WAS* (n = 97, 24.6%) were the most frequently mutated genes, followed by *CARMIL2* (n = 17, 4.3%), *STK4* (n = 13, 3.2%), *ARPC1B* (n = 12, 3.0%), and *DOCK2* (n = 11, 2.7%). Details are shown in Figure 2, with emphasis on the hotspot mutations in the MENA region. Moreover, Supplementary Figure S2, indicates frequencies of genetic diagnoses at a population level to indicate the proportion of each actinopathies based on geographical region. A genetic diagnosis was readily identified in patients with multiple cases in their family, representing 59.6% of all cases. Specific genetic defects associated with higher mortality rates and significantly decreased survival included DOCK8 (20.5%), ARPC1B (8.3%), DOCK2 (45.4%) and WASP (19.5%) deficiencies (Figure 3). Exonic mutations predominated (85.5%) over splicing mutations (14.5%); Type of mutations included 27.8% missense, 26.4% large deletion, 19.1% frameshift, 14.5% essential-splicing, and 11.9% stop gain nonsense mutations. Considering mild vs. severe variants (defined in the method section), there was no clear genotype-phenotype correlation in our cohort of actinopathies (Figure 3). However, severe genotypes correlated with severe phenotypes and higher mortality in certain genetic defects, mainly in the WAS (*p* = 0.01) and DOCK8 (*p = 0.05*) deficiencies (Supplementary Figure S3). Homozygous mutations were documented in 69.7%, hemizygous in 28.5%, heterozygous in 1.3%, and compound heterozygous in 0.5% of cases. The maternal carrier state was positive in 98% of the patients with X-linked recessive inheritance. Based on the defective GTPase pathway, 341 patients were assigned to the defective CDC42 pathway and 51 patients to the defective RAC2 pathway, with a higher rate of autosomal recessive pattern in the RAC2 GTPase group (80% vs. 69%, Figure 2). Only two patients were allocated to transcription/elongation of actin due to mutation in *WDR1* and *CEBPE* with almost similar survival rates compared to the remaining patients (Supplementary Figure S4). Supplementary Table S7, summarizes the clinical and immunologic profiling of the main six frequent (>10 patients) monogenic actinopathies. The lowest rate of infection was observed in STK4 deficiency (53.8%) and lower respiratory tract infections in CARMIL3 deficiency (23.5%). Dermatologic infections and atopic presentations were indicators of DOCK8 deficiency (70.2% and 94.8%, respectively) and autoimmunity and hematologic disorders were frequently observed in WAS deficiency (51.5% and 100%, respectively). Lymphoproliferation and malignancy were also prevalent among STK4 deficiency (76.9% and 23.0%, respectively). These patients also presented with leukopenia (median 2,790/ul) and lymphopenia (median 780/ul) mainly due to a reduction of T cells compared to other actinopathies. Low serum IgG levels and CD4^+^ T lymphopenia were common among monogenic actinopathies, however, B cell and NK cell lymphopenia were predominant in STK4- (median: 75/ul) and DOCK2- (median: 74/ul) deficient patients, respectively. Eosinophilia and increased serum IgE levels were identified in all monogenic actinopathies but with the highest counts among DOCK8 deficiency (median: 3,300/ul and 2000 IU/mL, respectively). The lowest level of MPV and IgM levels were detected in WASP deficiency (median: 7.1 fL and 24 mg/dL, respectively) (Supplementary Table S7).

### Clinical and molecular characteristics of the CDC42 GTPase subgroup

In total, 341 participants were enrolled in this group covering the following individual gene defects: *DOCK8, WAS, ARPC1B, CORO1A, PSTPIP1, WIPF1, MSN*. The majority of the affected patients were male (64.8%, Table 1; Supplementary Table S8). Turkish ethnicity was the most prevalent (37.2%), followed by Persian (34.6%). The median age of the participants at the commencement of the study was 107.5 months (0–372). The median age at disease onset was 4.5 months (0–270), while the median age at diagnosis was 36 months (0.1–300), demonstrating earlier onset of symptoms compared to the RAC2 GTPase group (*p* < 0.001) with a median delay in diagnosis of 25.5 months (0–264). The overall mortality rate in this group was 19.6%, highlighting a more severe rate of complications in the CDC42 GTPase group compared to the RAC2 GTPase group (*p* = 0.01). The median age at onset of eczema (6 [1–144] months), recurrent infections presentation (6 [0.1–192] months) and development of other atopic diseases (8 [1–96] months) was lower compared to the onset of other manifestations.

Recurrent infections were the most frequently reported presenting symptoms, accounting for 92.0% of the group, with allergic disease closely behind, at 77.1% (Table 2). The most frequent infections involved skin (54.2%), similar to lower RTI (51.3%). Viruses were the predominant organisms identified in these patients (68.7%), followed by Gram-positive (40.5%) and Gram-negative (32.1%) bacterial infections. Among the viruses, herpesviridae family (36.6%), mainly cytomegalovirus (CMV), was the most prevalent (29.8%) organism. Severe systemic infections including septicemia, and disseminated intravascular coagulation emerged as the predominant infectious complications of CDC42 GTPase patients compared to the RAC2 GTPase group (Table 2; Supplementary Table S4). Moreover, allergic presentations, particularly eczema (72.7%) were significantly more prevalent in the defective CDC42 pathway rather than RAC2 pathway (Table 2; Supplementary Table S5).

Genetic analysis revealed that *DOCK8* (62.1%) and *WAS* (28.4%) were the most frequently mutated genes in this group. Large deletion mutations were found in 29.9%, followed by missense mutations (25.3%). Homozygous mutations were documented in 69%, followed by hemizygous mutations in 29%. Most patients at the time of diagnosis had normal lymphocyte counts (67.3% of patients), normal CD8^+^ T cells (44.1%), normal B cells (65.7%), normal NK cells (65.7%), normal neutrophil counts (77%) normal platelet counts (48.5%), normal MPV (59%), normal IgG (60.9%) and normal IgA (56.9%), but low IgM (52.9%), high IgE (79.7%), and low CD3^+^ T cells (55.9%), mainly due to low CD4^+^ T cells (66.3%). Compared to the RAC2 GTPase group, most patients with defects in CDC42 pathway had a lower percentage of CD4^+^ (27.1% vs. 48.6% of total lymphocytes, *p* = 0.02) and of CD8^+^ (22.3% vs. 50.0% of total lymphocytes, *p* = 0.02) T cells and higher serum levels of IgE (3,414.3 vs. 213.1 IU/mL, *p* = 0.01, Table 3).

Of the CDC42 GTPase patients, 104 (30.4%) underwent HSCT, which was a significantly larger proportion than in the other groups (*p* < 0.001). The median age at transplantation was 60 (3–303) months, significantly lower than in the other groups (*p* = 0.007). The majority of patients (45.5%) received grafts from matched unrelated donors, while 28.6% received grafts from matched sibling donors. The primary source of HSCs was bone marrow, which accounted for 78.5% of cases, while peripheral blood derived stem cells were utilized in 20% of cases and cord blood in one patient. The conditioning regimen commonly used consisted of Fludarabine and Busulfan, accounting for 19.2% of cases. Four patients experienced rejection of the transplant, and 7 patients died post-transplantation. However, overall survival of HSCT patients was significantly improved compared to non-transplanted cases (*p* = 0.0001). Stratification of genetic data indicated that patients with defects in *DOCK8* (*p* < 0.0001) and *WAS* (*p* = 0.045) benefited from HSCT. Still, the limited experiences of HSCT in ARPC1B deficient patients in the MENA region were not similarly successful (Supplementary Figure S5).

### Clinical and molecular characteristics of the RAC2 GTPase subgroup

Fifty-one participants were enrolled in this group, covering the following individual gene defects: *CARMIL2, STK4, DOCK2, RAC2, RASGRP1, NCKAP1L.* The proportion of female patients was higher than in the CDC42 subgroup (52.9% vs., 35.2%, *p* = 0.014, Table 1; Supplementary Table S8). Persian ethnicity was the most prevalent (43.1%), followed by Turkish (31.1%). The median age of symptom onset and diagnosis was 29 (1–216) and 132 (5–264) months, respectively and was notably greater compared to the remaining groups (*p* < 0.001), with most patients displaying symptoms and being diagnosed during early adolescence (Supplementary Table S1). A positive family history of autoimmunity was reported in 40% of cases and was significantly higher than that of other groups. The overall survival rate in this group was 94.2%, corresponding to the late-onset of symptoms and to better clinical management in the MENA region, despite a median delay in diagnosis of 97 (0.1–207) months.

The age at first manifestation was higher for all complications in patients with RAC2 pathway defects except for malignancy than in the comparison group (Table 1). The median age at onset of recurrent infections (30 [3–120] months) and eczema (31 [3–168] months) was lower compared to the other manifestations. The highest median age at disease onset was observed in lymphoproliferation, 132 (48–228) months. Infectious presentations were the most frequently reported complication, accounting for 74.5% in the RAC2 group, and were mainly due to recurrent sinusitis and dermatologic infections (Table 2; Supplementary Tables S2, S3). Similar to the CDC42 GTPase group, viruses were the most common agents, exhibiting a greater frequency of Epstein–Barr virus (EBV) (29.4%) and HPV (17.2%) infection. Moreover, Gram-negative bacteria were more frequent than Gram-positive ones. While allergic features were slightly less frequent in the RAC2 GTPase patients (52.8%, particularly with asthma diagnosis), 35.3% of the individuals displayed at least one lymphoproliferative manifestation, which was significantly higher than that of the other groups (*p* = 0.001). The main recorded presentations in this category were lymphadenopathy (34.5%, Supplementary Table S5). Evans syndrome and psoriasis were among the most common autoimmune clinical presentations in the RAC2 GTPase group compared to the CDC42 GTPase group. High levels of type I and type II interferons were also detected in the RAC2 GTPase group (Supplementary Table S6).

Molecular analysis revealed that *CARMIL2* and *STK4* were the genes with the highest mutation rate, at 33.3% and 25.4%, respectively. Most patients showed missense variants (47.5%), followed by stop-gain nonsense mutations (22.5%). Homozygous mutations were documented in 80%, while hemizygous mutations in 18% of patients (Figure 2). In agreement with the clinical phenotype and decreased mortality, RAC2 GTPase patients had almost normal immunologic profiling (except for low CD4^+^ T cells in 48.4%) at the time of diagnosis suggesting more functional than developmental defects in this group. Normal absolute lymphocyte counts were documented in the majority of patients (56.3%) as well as normal CD3^+^ T cells (41.9%), CD8 + T cells (35.5%), CD19^+^ B cells (51.6%), NK cells (63%), neutrophil counts (75%), platelet counts (93.8%), MPV (81%), IgG (65.6%), IgM (62.5%), IgA (70%) and IgE serum levels (56.5%). In comparison to the CDC42 group, they had less leukocytosis (*p* < 0.001), and normal counts of neutrophils (*p* = 0.04) and B cells (*p* = 0.02, Table 3). Only 5 patients (9.8%) underwent HSCT. Overall, 80% received a matched unrelated graft while 20% received a matched sibling graft. All 5 patients are fully immune reconstituted. HSCT in DOCK2 deficient patients, in particular, resulted in a significantly higher survival rate than non-transplanted cases (*p* = 0.015, Supplementary Figure S5).

### Transcription/elongation group

There were only two individuals within this cohort, one female and one male, one of Turkish descent and the other coming from Qatar. Disease manifestation occurred during infancy, with one individual receiving a diagnosis during middle childhood and the other during early adolescence, after a considerable delay of 102 months. Omphalitis and postauricular lymphadenitis observed in the neonatal period, were the first manifestations of one patient, while neutropenia, thrombocytopenia and failure to thrive occurred in the second patient as the first presentation. Both patients exhibited infectious presentations, with one patient experiencing cytopenia. One patient was found to have a homozygous missense mutation in *WDR1,* while another had a homozygous frameshift mutation in *CEBPE* (Supplementary Table S8). The latter patient showed a normal complete blood count with normal MPV, low IgA, high IgG, high IgM, and normal IgE. None of the two patients were transplanted and both are alive.

## Discussion

The MENA region encompasses 22 countries and a diverse population with a significant prevalence of consanguinity. In this geographic area, we encounter a higher incidence of syndromic combined immune deficiencies compared to American and European countries. The heightened prevalence of autosomal recessive diseases is associated with the common culture of related marriages in the MENA region. Genetic investigations conducted in this geographical area have uniquely contributed to our general understanding of autosomal recessive disorders and to the identification of novel genes and molecular pathways. In line with this notion, most of the genes responsible for actin-related IEIs except for WAS have been discovered by studying cohort of patients from MENA countries. For the current study we identified 503 patients with different genetic defects with a broad disease spectrum and disease severity and prognosis. A literature review of actine-related IEIs showed that only diseases affecting the CDC42 GTPase pathway, namely WAS and DOCK8, have been reported in large enough numbers to allow a comparative analysis of different actinopathies. Moreover, the available evidence for the treatment of other rare actinopathies is scarce since the majority of Western IEI registries have mostly reported patients with WAS defects and other X-linked diseases. Still conversely, we observed dominantly DOCK8 defects and other autosomal recessive diseases as the main genetic defects in this region, indicating the need for the current detailed study on clinical, immunologic and molecular defects of actinopathies using MENA-IEI registry data.

About one-fifth of our current cohort were patients with the clinical diagnosis of WAS that were molecularly not defined with a more severe phenotype and higher mortality rate. Although patients enrolled in this study were 120 months of age on average, molecularly undefined patients only survived for 55 months with 42.2% mortality. Between affected patients with actinopathies, there can be significant variations in the penetrance of genetic defects, severity, and timing of the emergence of clinical symptoms (Albert et al., 2010; Imai et al., 2004). While some cases may have a normal life-span, with thrombocytopenia being the sole symptom, others may experience life-threatening opportunistic infections and bleeding in early infancy, and early infant mortality if treatment is not received (Mahlaoui et al., 2013). A large cohort of 577 WASP-deficient patients from 26 European and American countries has been recently evaluated and published (Vallee et al., 2024). Within this Western cohort, the average age of clinical diagnosis was 106 months with a survival rate of 84.4%, similar to our molecularly defined WAS patients. Although the rate of genetic analysis and availability of biobanking systems for post-mortem DNA sequencing affect this finding in specific countries in MENA region (e.g., Morocco), it also indicates the importance of finding biomarkers for the detection of this proportion of patients with actinopathies with complete penetrance and severe early manifestations for better clinical management and more intense treatment or HSCT prioritization.

Our study is unique in that it integrated patients across 15 characterized actin-related IEIs. Although we did not observe a clear genotype-phenotype correlation between patients with different types of actinopathies, specific genetic defects mainly from the CDC42 GTPase pathway including WAS and DOCK8 showed higher mortality in the context of severe genetic mutations. While a certain degree of genotype/phenotype connection has been demonstrated in actinopathies in other cohorts (Vallee et al., 2024; Aydin et al., 2015), prior research has shown that individuals with milder variations have a reduced chance of experiencing severe disease-associated outcomes (Imai et al., 2004; Zhu et al., 1997). If a patient has a qualified stem cell donor and has a characteristic severe actin-related phenotype in childhood, HSCT is the best available treatment option and is obviously warranted (Albert et al., 2022; Burroughs et al., 2020). Conversely, selecting the best course of action for patients with milder phenotypes and delayed diagnosis can be challenging when weighing risk versus benefit because the disease may worsen throughout a patient’s life, and even mild symptoms may have a substantial detrimental effect on quality of life (Shah et al., 2019). Despite the severity of mutation, our data from HSCT patients also highlighted the importance of this modality in CDC42 GTPase defects and specific disorders of RAC2 GTPase defects, particularly DOCK2 deficiency. Different therapeutic approaches are available for patients with actin-related IEI, ranging from symptomatic treatment, prophylactic application of antimicrobial agents or Ig-therapy, and thrombopoietic agents, to definitive therapeutic options such as HSCT or gene therapy (Worth and Thrasher, 2015). Antibiotics or Ig therapy may prevent many severe infections but will not eliminate the risk of bleeding, autoimmunity, or malignancy. HSCT has become increasingly successful and can completely cure the disease, but it carries a small but significant risk of mortality as well as short and long-term morbidity (Moratto et al., 2011). One of the main limitations of the current study is the inclusion of different exome capture kits and sequencing platforms which might impact the quality and outcomes of analysis. Although we properly normalized batches in our analysis different capture kits define target regions differently and vary in their ability to capture GC-rich or challenging regions. Moreover, patients were diagnosed at different time points and newer versions of capturing kits generally offer better coverage, uniformity and higher efficiency, which improves variant detection and reduces off-target reads (Belova et al., 2025). Sequencing platforms also vary in performance and using different sequencing chemistry, which potentially may exhibit unique error profiles and shorter read lengths, potentially affecting alignment and variant calling. These influences on read depth, coverage uniformity, and variant calling accuracy may affect the detection of rare, structural, or low-frequency variants, and complicate downstream analyses such as copy number variation detection and population-level comparisons (Lelieveld et al., 2015). To ensure robust and reproducible results, it is important in future to use consistent methodologies for actinopathies and validate key findings using orthogonal methods. Harmonized classification of mutations severity in different genes that all are matched with ACMG pathogenic criteria might be informative as shown in this study and other published papers previously (Salami et al., 2025; Amirifar et al., 2021), but other approaches should be considered as alternative. By specific gene identification and report of patients may specific domain analysis or computational algorithms or gene-specific evidence will be established for each gene associated with actinopathies. Ultimately functional assays and unform motility experiments provide strong support for variant interpretation (Fang et al., 2022).

Actinopathies, particularly WAS deficiency, were the first IEI to be cured by allogeneic HSCT (Bach et al., 1968). Although HSCT has grown in popularity and is now capable of curing the disease entirely, there remains a slight but substantial risk of death in addition to both short- and long-term morbidity. Nevertheless, actinopathies remain multifaceted, posing very complex challenges to physicians and families alike. The findings of our study showed that the affected pathway of GTPase and the severity of variation are reliable biomarkers that may be used to predict the prognosis of the disease in patients with actinopathies. Defects in RAC2 regulators of actin are usually diagnosed late due to a normal immune profile, but a higher rate of specific viruses (EBV and HPV), gastrointestinal autoimmunity, asthma, and lymphoproliferation compared to CDC42 regulatory defects.

## Conclusion

Majority of patients from MENA-IEI registry with actinopathies have defects in CDC42 GTPase and RAC2 GTPase pathways. Defects in the actin recycling/transcription sub-branch were extremely rare in our cohort, and we cannot enter this group into our pathway analysis due to lack of statistical power, therefore this group should be more studied in the future. Although patients with less severe genetic variations may generally anticipate a delayed start of severe complications, they are nevertheless at risk for morbidity and early death, which supports the investigation of early decisive treatment in this subset of actin-related IEI. These results will aid in improving the counseling provided to relatives of patients with actinopathies.

## Data Availability

The original contributions presented in the study are included in the article/Supplementary Material, further inquiries can be directed to the corresponding authors.
