# Supplementary material for: Clinical and molecular findings in actin-related inborn errors of immunity: the middle East and North Africa registry
Source: Front Genet. 2025 Aug 8;16:1584681. doi: 10.3389/fgene.2025.1584681 (PMC12370701; doi:10.3389/fgene.2025.1584681)
Supplement: Supplementary file 1 [file DataSheet1.docx]

**Supplementary file-**

**Clinical and Molecular Findings in Actin-related Inborn Errors of Immunity: The Middle East and North Africa Registry**

**Table S1. Age distribution of 503 patients with actin-related inborn errors of immunity at the time of onset, diagnosis, and during the current study.**

| **Parameters** | **Total**  **(n=503)** | **Unsolved**  **(n=109)** | **Other actinopathies**  **(n=2)** | **CDC42 pathway**  **(n=347)** | **RAC2 pathway**  **(n=45)** | ***P*-value** |
| --- | --- | --- | --- | --- | --- | --- |
| Age at study, categorized, (n=246) | | | | | | |
| *Neonate (0-1 months)* | 2(0.4) | 0(0) | 0(0) | 2(0.6) | 0(0) | 0.186 |
| *Infancy (1-12 months)* | 61(12.1) | 6(6) | 0(0) | 48(13.8) | 4(8.9) |  |
| *Toddler (13-24 months)* | 19(3.8) | 10(9) | 0(0) | 11(3.2) | 0(0) |  |
| *Early Childhood (25-60 months)* | 65(12.9) | 26(24) | 0(0) | 42(12.1) | 2(4.4) |  |
| *Middle Childhood (61-132 months)* | 149(29.6) | 10(9) | 0(0) | 120(34.6) | 10(22.2) |  |
| *Early Adolescence (133-216 months)* | 129(25.6) | 35(32) | 2(100) | 79(22.8) | 16(35.6) |  |
| *Late Adolescence (217- 252 months)* | 31(6.2) | 10(9) | 0(0) | 17(4.9) | 6(13.3) |  |
| *Adults (>253 months)* | 47(9.3) | 13(12) | 0(0) | 28(8.1) | 8(17.8) |  |
| Age at onset, categorized, (n=346) | | | | | | |
| *Neonate (0-1 months)* | 139(27.6) | 39(36) | 1(50) | 94(27.1) | 3(6.7) | <0.001* |
| *Infancy (1-12 months)* | 258(51.3) | 60(55) | 1(50) | 182(52.4) | 13(28.9) |  |
| *Toddler (13-24 months)* | 31(6.2) | 4(4) | 0(0) | 23(6.6) | 5(11.1) |  |
| *Early Childhood (25-60 months)* | 49(9.7) | 4(4) | 0(0) | 32(9.2) | 16(35.6) |  |
| *Middle Childhood (61-132 months)* | 20(4) | 0(0) | 0(0) | 15(4.3) | 6(13.3) |  |
| *Early Adolescence (133-216 months)* | 4(0.8) | 3(3) | 0(0) | 0(0) | 2(4.4) |  |
| *Late Adolescence (217- 252 months)* | 0(0) | 0(0) | 0(0) | 0(0) | 0(0) |  |
| *Adults (>253 months)* | 2(0.4) | 0(0) | 0(0) | 1(0.3) | 0(0) |  |
| Age at diagnosis, categorized, (n=330) | | | | | | |
| *Neonate (0-1 months)* | 37(7.4) | 12(11) | 0(0) | 23(6.6) | 0(0) | <0.001* |
| *Infancy (1-12 months)* | 143(28.4) | 49(45) | 0(0) | 87(25.1) | 1(2.2) |  |
| *Toddler (13-24 months)* | 49(9.7) | 15(14) | 0(0) | 30(8.6) | 3(6.7) |  |
| *Early Childhood (25-60 months)* | 113(22.5) | 21(19) | 0(0) | 86(24.8) | 8(17.8) |  |
| *Middle Childhood (61-132 months)* | 113(22.5) | 9(8) | 2(100) | 96(27.7) | 12(26.7) |  |
| *Early Adolescence (133-216 months)* | 35(7) | 4(4) | 0(0) | 17(4.9) | 15(33.3) |  |
| *Late Adolescence (217- 252 months)* | 8(1.6) | 0(0) | 0(0) | 5(1.4) | 1(2.2) |  |
| *Adults (>253 months)* | 5(1) | 0(0) | 0(0) | 3(0.9) | 1(2.2) |  |
| *M; male, F; female, D; dead, A; alive, IQR; interquartile range. *A comparison between CDC42 and RAC2 groups performed and p-value<0.05 is considered as significant.* | | | | | | |

**Table S2. Details of respiratory tract infectious complications in 340 cases out of 503 patients with actin-related inborn errors of immunity.**

| **Infectious presentations (organ based)** | **Total**  **(n=340)** | **Unsolved**  **(n=78)** | **Other actinopathies**  **(n=2)** | **CDC42 pathway**  **(n=231)** | **RAC2 pathway**  **(n=29)** | **P-value** |
| --- | --- | --- | --- | --- | --- | --- |
| Recurrent otitis media /otitis media with effusion, n (%) | 106 (31.2) | 17 (21.8) | 1 (50) | 79 (34.2) | 9 (31) | 0.99 |
| Extern otitis, n (%) | 2 (0.6) | 0 (0) | 0 (0) | 2 (0.9) | 0 (0) | 0.97 |
| Chronic otorrhea, n (%) | 1 (0.3) | 0 (0) | 0 (0) | 1 (0.4) | 0 (0) | 0.99 |
| Recurrent sinusitis, n (%) | 47 (13.8) | 16 (23.5) | 0 (0) | 23 (10) | 8 (27.6) | 0.04* |
| Chronic cough, n (%) | 5 (1.5) | 2 (2.6) | 0 (0) | 3 (1.3) | 0 (0) | 0.94 |
| Mastoiditis, n (%) | 2 (0.6) | 0 (0) | 0 (0) | 2 (0.9) | 0 (0) | 0.97 |
| Otomastoiditis, n (%) | 1 (0.3) | 0 (0) | 0 (0) | 1 (0.4) | 0 (0) | 0.99 |
| Dental abscess, n (%) | 4 (1.2) | 1 (1.3) | 0 (0) | 3 (1.3) | 0 (0) | 0.94 |
| Perichondritis, n (%) | 1 (0.3) | 1 (1.3) | 0 (0) | 0 (0) | 0 (0) | 1.00 |
| Tonsilitis, n (%) | 2 (0.6) | 0 (0) | 0 (0) | 2 (0.9) | 0 (0) | 0.97 |
| Peritonsillar abscess, n (%) | 1 (0.3) | 0 (0) | 0 (0) | 1 (0.4) | 0 (0) | 0.99 |
| Nasopharyngitis, n (%) | 49 (14.4) | 3 (3.8) | 1 (50) | 43 (18.6) | 2 (6.9) | 0.48 |
| Pneumonia, n (%) | 152 (44.7) | 23 (29.5) | 1 (50) | 117 (50.6) | 11 (37.9) | 0.64 |
| Other lower respiratory tract infection, n (%) | 62 (18.2) | 29 (37.2) | 0 (0) | 27 (11.7) | 6 (20.7) | 0.60 |
| Bronchiectasis | 41 (12.1) | 0 (0) | 1 (50) | 36 (15.6) | 4 (13.8) | 1.00 |
| Bronchiolitis, n (%) | 22 (6.5) | 0 (0) | 0 (0) | 22 (9.5) | 0 (0) | 0.39 |
| Bronchitis, n (%) | 4 (1.2) | 0 (0) | 1 (50) | 2 (0.9) | 1(3.4) | 0.68 |
| Empyema, n (%) | 2 (0.6) | 0 (0) | 0 (0) | 2 (0.9) | 0 (0) | 0.97 |
| Pneumatocele, n (%) | 1 (0.3) | 0 (0) | 0 (0) | 1 (0.4) | 0 (0) | 0.99 |
| Lung abscess, n (%) | 2 (0.6) | 0 (0) | 0 (0) | 1 (0.4) | 1(3.4) | 0.38 |
| Pneumothorax, n (%) | 1 (0.3) | 0 (0) | 0 (0) | 1 (0.4) | 0 (0) | 0.99 |
| Atelectasis, n (%) | 1 (0.3) | 0 (0) | 0 (0) | 1 (0.4) | 0 (0) | 0.99 |
| *Pneumocystis jirovecii* pneumonia, n (%) | 3 (0.9) | 0 (0) | 0 (0) | 3 (1.3) | 0 (0) | 0.94 |
| Pulmonary tuberculosis, n (%) | 2 (0.6) | 0 (0) | 0 (0) | 2 (0.9) | 0 (0) | 0.97 |
| CMV pneumonitis, n (%) | 1 (0.3) | 0 (0) | 0 (0) | 1 (0.4) | 0 (0) | 0.99 |
| Severe COVID-19 pneumonia, n (%) | 3 (0.9) | 0 (0) | 0 (0) | 3 (1.3) | 0 (0) | 0.94 |
| ** A comparison between CDC42 and RAC2 groups performed and p-value<0.05 is considered as significant.*  *CMV: Cytomegalovirus, COVID-19: Coronavirus disease 2019.* | | | | | | |

**Table S3. Details of dermatologic and mucosal infectious complications in 340 cases out of 503 patients with actin-related inborn errors of immunity.**

| **Infectious presentations (organ based)** | **Total**  **(n=340)** | **Unsolved**  **(n=78)** | **Other actinopathies**  **(n=2)** | **CDC42 pathway**  **(n=231)** | **RAC2 pathway**  **(n=29)** | ***P*-value** |  |
| --- | --- | --- | --- | --- | --- | --- | --- |
| Skin bacterial infections, n (%) | 31 (9.1) | 6 (7.7) | 0 (0) | 24 (10.4) | 1(3.4) | 0.70 |  |
| *Cellulitis, n (%)* | 6 (1.8) | 2 (2.6) | 1 (50) | 3 (1.3) | 0 (0) | 0.94 |  |
| *Folliculitis and furuncles, n (%)* | 2 (0.6) | 0 (0) | 0 (0) | 0 (0) | 2 (6.9) | <0.001* |  |
| *Skin ulcer, n (%)* | 2 (0.6) | 0 (0) | 0 (0) | 0 (0) | 2 (6.9) | <0.001* |  |
| *Recurrent vesiculopustular lesion, n (%)* | 1 (0.3) | 0 (0) | 0 (0) | 1 (0.4) | 0 (0) | 0.99 |  |
| *Paronychia, n (%)* | 1 (0.3) | 0 (0) | 0 (0) | 1 (0.4) | 0 (0) | 0.99 |  |
| *Skin papulopustular infection, n (%)* | 2 (0.6) | 0 (0) | 0 (0) | 2 (0.9) | 0 (0) | 0.97 |  |
| *Impetigo, n (%)* | 3 (0.9) | 0 (0) | 0 (0) | 3 (1.3) | 0 (0) | 0.94 |  |
| *Omphalitis, n (%)* | 1 (0.3) | 0 (0) | 1 (50) | 0 (0) | 0 (0) | 1.00 |  |
| *Pyoderma, n (%)* | 1 (0.3) | 1 (1.3) | 0 (0) | 0 (0) | 0 (0) | 1.00 |  |
| *Skin abscess, n (%)* | 22 (6.5) | 1 (1.3) | 1 (50) | 18 (7.8) | 2 (6.9) | 1.00 |  |
| *Perianal abscess, n (%)* | 3 (0.9) | 1 (1.3) | 0 (0) | 2 (0.9) | 0 (0) | 0.97 |  |
| *Finger abscess, n (%)* | 2 (0.6) | 1 (1.3) | 0 (0) | 1 (0.4) | 0 (0) | 0.99 |  |
| *Stomatitis, n (%)* | 10 (2.9) | 0 (0) | 1 (50) | 9 (3.9) | 0 (0) | 0.76 |  |
| *Gingivitis, n (%)* | 6 (1.8) | 2 (2.6) | 2 (100) | 2 (0.9) | 0 (0) | 0.97 |  |
| *BCGitis, n (%)* | 8 (2.4) | 2 (2.6) | 1 (50) | 5 (2.2) | 0 (0) | 0.89 |  |
| *BCG lymphadenitis, n (%)* | 3 (0.9) | 0 (0) | 1 (50) | 2 (0.9) | 0 (0) | 0.97 |  |
| *Local BCG suppurative abscess, n (%)* | 1 (0.3) | 0 (0) | 0 (0) | 1 (0.4) | 0 (0) | 0.99 |  |
| Skin viral infections, n (%) | 242(25.7) | 35(32.1) | 2 (100) | 185(53.3) | 20(44.8) | 0.59 |  |
| *MCV infection, n (%)* | 12 (3.5) | 0 (0) | 0 (0) | 10 (4.3) | 2 (6.9) | 0.94 |  |
| *Skin HSV infection, n (%)* | 9 (2.6) | 1 (1.3) | 0 (0) | 8 (3.5) | 4 (13.8) | 0.10 |  |
| *Herpes labial infection, n (%)* | 7 (2.1) | 0 (0) | 0 (0) | 3 (1.3) | 4 (13.8) | <0.001* |  |
| *Oral HSV infection, n (%)* | 5 (1.5) | 0 (0) | 0 (0) | 5 (2.2) | 0 (0) | 0.89 |  |
| *Herpetic perioral lesions, n (%)* | 2 (0.6) | 0 (0) | 0 (0) | 2 (0.9) | 0 (0) | 0.97 |  |
| *Eczema herpeticum, n (%)* | 3 (0.9) | 0 (0) | 0 (0) | 3 (1.3) | 0 (0) | 0.94 |  |
| *Recurrent HSV infection, n (%)* | 2 (0.6) | 0 (0) | 0 (0) | 2 (0.9) | 0 (0) | 0.97 |  |
| *Kaposi-Juliusberg’s syndrome, n (%)* | 1 (0.3) | 0 (0) | 0 (0) | 1 (0.4) | 0 (0) | 0.99 |  |
| *Skin VZV infection, n (%)* | 8 (2.3) | 1 (1.3) | 0 (0) | 6 (2.5) | 1(3.4) | 1.00 |  |
| *Wart, n (%)* | 10 (2.9) | 0 (0) | 0 (0) | 5 (2.2) | 5 (17.2) | <0.001* |  |
| *Other HPV infection, n (%)* | 5 (1.5) | 0 (0) | 0 (0) | 5 (2.2) | 0 (0) | 0.89 |  |
| *Genital HPV infection, n (%)* | 2 (0.6) | 0 (0) | 0 (0) | 2 (0.9) | 0 (0) | 0.97 |  |
| *Epidermodysplasia verruciformis, n (%)* | 2 (0.6) | 0 (0) | 0 (0) | 2 (0.9) | 0 (0) | 0.97 |  |
| *Hand, foot and mouth disease, n (%)* | 1 (0.3) | 0 (0) | 0 (0) | 1 (0.4) | 0 (0) | 0.99 |  |
| Skin fungal infections, n (%) | 47 (13.8) | 8 (10.3) | 1 (50) | 37 (16) | 1(3.4) | 0.35 |  |
| *Candidiasis, n (%)* | 47 (13.8) | 8 (10.3) | 1 (50) | 37 (16) | 1(3.4) | 0.35 |  |
| *Onychomycosis/fungal nail infection, n (%)* | 2 (0.6) | 0 (0) | 0 (0) | 2 (0.9) | 0 (0) | 0.97 |  |
| Other mucosal infections, n (%) | 90 (26.5) | 33 (42.3) | 1 (50) | 50 (21.6) | 6 (20.7) | 1.00 |  |
| *Diarrhea, n (%)* | 37 (10.9) | 18 (23.1) | 1 (50) | 15 (6.5) | 3 (10.3) | 0.90 |  |
| *Chronic diarrhea, n (%)* | 15 (4.4) | 4 (5.1) | 0 (0) | 10 (4.3) | 1(3.4) | 1.00 |  |
| *Bloody diarrhea, n (%)* | 5 (1.5) | 2 (2.6) | 0 (0) | 3 (1.3) | 0 (0) | 0.94 |  |
| *Gastroenteritis, n (%)* | 28 (8.2) | 9 (11.5) | 0 (0) | 17 (7.4) | 2 (6.9) | 1.00 |  |
| *Urinary tract infections, n (%)* | 14 (4.1) | 6 (7.7) | 1 (50) | 7 (3) | 0 (0) | 0.82 |  |
| ** A comparison between CDC42 and RAC2 groups performed and p-value<0.05 is considered as significant.*  *HSV, Herpes simplex virus, VZV, Varicella zoster virus , MCV,* *Molluscum contagiosum virus, HPV,* *Human papillomavirus, BCG, Bacillus Calmette–Guérin* | | | | | | | |

**Table S4. Details of severe infectious complications in 340 cases out of 503 patients with actin-related inborn errors of immunity.**

| **Infectious presentations (organ based)** | **Total**  **(n=340)** | **Unsolved**  **(n=78)** | **Other actinopathies**  **(n=2)** | **CDC42 pathway**  **(n=231)** | **RAC2 pathway**  **(n=29)** | ***P*-value** |
| --- | --- | --- | --- | --- | --- | --- |
| Urethritis, n (%) | 1 (0.3) | 1 (1.3) | 0 (0) | 0 (0) | 0 (0) | 1.00 |
| Renal abscess, n (%) | 4 (1.2) | 0 (0) | 0 (0) | 4 (1.7) | 0 (0) | 0.92 |
| Liver infectious presentations, n (%) | 9 (2.6) | 0 (0) | 0 (0) | 9 (3.9) | 0 (0) | 0.76 |
| Liver abscess, n (%) | 5 (1.5) | 0 (0) | 0 (0) | 5 (2.2) | 0 (0) | 0.89 |
| Nervous system infections, n (%) | 16 (4.7) | 3 (3.8) | 0 (0) | 12 (5.2) | 1(3.4) | 0.98 |
| *Meningitis, n (%)* | 11 (3.2) | 3 (3.8) | 0 (0) | 7 (3) | 1(3.4) | 1.00 |
| *Encephalitis, n (%)* | 3 (0.9) | 0 (0) | 0 (0) | 3 (1.3) | 0 (0) | 0.94 |
| *Meningoencephalitis, n (%)* | 2 (0.6) | 0 (0) | 0 (0) | 2 (0.9) | 0 (0) | 0.97 |
| Joint and bone infections, n (%) | 9 (2.6) | 2 (2.6) | 0 (0) | 7 (3) | 0 (0) | 0.82 |
| *Septic arthritis, n (%)* | 2 (0.6) | 0 (0) | 0 (0) | 2 (0.9) | 0 (0) | 0.97 |
| *Osteomyelitis, n (%)* | 4 (1.2) | 0 (0) | 0 (0) | 4 (1.7) | 0 (0) | 0.92 |
| *Myositis, n (%)* | 1 (0.3) | 0 (0) | 0 (0) | 1 (0.4) | 0 (0) | 0.99 |
| *Arthritis, n (%)* | 2 (0.6) | 2 (2.6) | 0 (0) | 0 (0) | 0 (0) | 1.00 |
| Ophthalmic infections, n (%) | 10 (2.9) | 0 (0) | 1 (50) | 7 (3) | 2 (6.9) | 0.76 |
| *Conjunctivitis, n (%)* | 4 (1.2) | 0 (0) | 1 (50) | 2 (0.9) | 1(3.4) | 0.68 |
| *Keratitis, n (%)* | 1 (0.3) | 0 (0) | 0 (0) | 1 (0.4) | 0 (0) | 0.99 |
| *CMV retinitis, n (%)* | 1 (0.3) | 0 (0) | 0 (0) | 1 (0.4) | 0 (0) | 0.99 |
| *Cataract due to CMV, n (%)* | 1 (0.3) | 0 (0) | 0 (0) | 1 (0.4) | 0 (0) | 0.99 |
| *Episcleritis, n (%)* | 1 (0.3) | 0 (0) | 0 (0) | 1 (0.4) | 0 (0) | 0.99 |
| *HSV conjunctivitis, n (%)* | 2 (0.6) | 0 (0) | 0 (0) | 1 (0.4) | 1(3.4) | 0.38 |
| Sepsis/ DIC, n (%) | 18 (5.3) | 6 (7.7) | 0 (0) | 12 (5.2) | 0 (0) | 0.66 |
| Catheter infection, n (%) | 1 (0.3) | 0 (0) | 0 (0) | 1 (0.4) | 0 (0) | 0.99 |
| *CMV viremia, n (%)* | 20 (5.9) | 1 (1.3) | 0 (0) | 19 (8.2) | 0 (0) | 0.46 |
| *Intrauterine CMV infection, n (%)* | 1 (0.3) | 0 (0) | 0 (0) | 1 (0.4) | 0 (0) | 0.99 |
| *Disseminated CMV, n (%)* | 1 (0.3) | 0 (0) | 0 (0) | 1 (0.4) | 0 (0) | 0.99 |
| *EBV viremia, n (%)* | 9 (2.6) | 1 (1.3) | 0 (0) | 7 (3) | 1(3.4) | 1.00 |
| *Disseminated VZV, n (%)* | 1 (0.3) | 0 (0) | 0 (0) | 1 (0.4) | 0 (0) | 0.99 |
| *Disseminated HSV infection, n (%)* | 1 (0.3) | 0 (0) | 0 (0) | 1 (0.4) | 0 (0) | 0.99 |
| *Other type of viremia, n (%)* | 4 (1.2) | 0 (0) | 0 (0) | 4 (1.7) | 0 (0) | 0.92 |
| *Military tuberculosis, n (%)* | 2 (0.6) | 0 (0) | 0 (0) | 0 (0) | 2 (6.9) | <0.001* |
| *Disseminated BCG infection, n (%)* | 1 (0.3) | 0 (0) | 0 (0) | 1 (0.4) | 0 (0) | 0.99 |
| *Bacteremia, n (%)* | 3 (0.9) | 0 (0) | 0 (0) | 3 (1.3) | 0 (0) | 0.94 |
| Nonspecific severe infections, n (%) | 15 (4.4) | 4 (5.1) | 1 (50) | 9 (3.9) | 1(3.4) | 1.00 |
| Chronic fever, n (%) | 7 (2.1) | 1 (1.3) | 0 (0) | 6 (2.6) | 0 (0) | 0.86 |
| Growth retardation/ failure to thrive, n (%) | 4 (1.2) | 1 (1.3) | 1 (50) | 2 (0.9) | 0 (0) | 0.97 |
| Maternal-fetal infection, n (%) | 1 (0.3) | 1 (1.3) | 0 (0) | 0 (0) | 0 (0) | 1.00 |
| ** A comparison between CDC42 and RAC2 groups performed and p-value<0.05 is considered as significant.*  *HSV, Herpes simplex virus, VZV, Varicella zoster virus, CMV: Cytomegalovirus, EBV: Epstein–Barr virus, DIC, Disseminated intravascular coagulation, BCG, Bacillus Calmette–Guérin* | | | | | | |

**Table S5**: **Details of immunological and hematological non-infectious complications in 503 patients with actin-related inborn errors of immunity.**

| **Parameters** | **Total**  **(n=340)** | **Unsolved**  **(n=78)** | **Other actinopathies**  **(n=2)** | **CDC42 pathway**  **(n=231)** | **RAC2 pathway**  **(n=29)** | ***P*-value** |  |
| --- | --- | --- | --- | --- | --- | --- | --- |
| Eczema, n (%) | 231(67.9) | 51(65.4) | 0 (0) | 168(72.7) | 12(41.4) | 0.01* |  |
| *Food allergy, n (%)* | 53(15.6) | 0(0) | 0 (0) | 53(22.9) | 2(6.9) | 0.26 |  |
| *Asthma, n (%)* | 53(15.6) | 0(0) | 0 (0) | 40(17.3) | 14(48.3) | <0.001* |  |
| *Conjunctivitis, n (%)* | 3(0.9) | 0(0) | 0 (0) | 4(1.7) | 0(0) | 0.92 |  |
| *Allergic rhinitis, n (%)* | 6(1.8) | 0(0) | 0 (0) | 4(1.7) | 3(10.3) | 0.06 |  |
| *Angioedema, n (%)* | 3(0.9) | 0(0) | 0 (0) | 3(1.3) | 0(0) | 0.94 |  |
| *Urticaria, n (%)* | 2(0.6) | 0(0) | 0 (0) | 2(0.9) | 0(0) | 0.97 |  |
| *Eosinophilic esophagitis, n (%)* | 1(0.3) | 0(0) | 0 (0) | 0(0) | 1(3.4) | 0.05 |  |
| *Mast cell activation syndrome, n (%)* | 1(0.3) | 1(1.3) | 0 (0) | 0(0) | 0(0) | 1.00 |  |
| Bruises and Ecchymosis, n (%) | 60(17.6) | 17(21.8) | 0 (0) | 40(17.3) | 3(10.3) | 0.82 |  |
| *Bleeding, n (%)* | 39(11.5) | 11(14.1) | 0 (0) | 26(11.3) | 2(6.9) | 0.92 |  |
| *Bloody diarrhea/ blood in stool, n (%)* | 9(2.6) | 0(0) | 0 (0) | 8(3.5) | 1(3.4) | 1.00 |  |
| *Subarachnoid hemorrhage, n (%)* | 1(0.3) | 0(0) | 0 (0) | 1(0.4) | 0(0) | 0.99 |  |
| *Mucocutaneous hemorrhage, n (%)* | 1(0.3) | 1(1.3) | 0 (0) | 0(0) | 0(0) | 1.00 |  |
| *Hemorrhagic syndrome, n (%)* | 1(0.3) | 1(1.3) | 0 (0) | 0(0) | 0(0) | 1.00 |  |
| *Hematemesis, n (%)* | 1(0.3) | 0(0) | 0 (0) | 1(0.4) | 0(0) | 0.99 |  |
| *Epistaxis, n (%)* | 16(4.7) | 5(6.4) | 0 (0) | 11(4.8) | 0(0) | 0.70 |  |
| *Hematoma, n (%)* | 2(0.6) | 0(0) | 0 (0) | 2(0.9) | 0(0) | 0.97 |  |
| *Petechia/purpura, n (%)* | 26(7.6) | 11(14.1) | 0 (0) | 15(6.5) | 0(0) | 0.57 |  |
| *Persistent petechia, n (%)* | 1(0.3) | 1(1.3) | 0 (0) | 0(0) | 0(0) | 1.00 |  |
| *Neonatal purpura, n (%)* | 1(0.3) | 1(1.3) | 0 (0) | 0(0) | 0(0) | 1.00 |  |
| *Lack of factor VII, n (%)* | 1(0.3) | 0(0) | 0 (0) | 1(0.4) | 0(0) | 0.99 |  |
| Cytopenia, n (%) | 145(42.6) | 65(83.3) | 1 (50) | 71(30.7) | 8(27.6) | 0.99 |  |
| *Thrombocytopenia, n (%)* | 114(33.5) | 64(82.1) | 1 (50) | 48(20.8) | 1(3.4) | 0.17 |  |
| *Anemia, n (%)* | 22(6.5) | 0(0) | 0 (0) | 16(6.9) | 6(20.7) | 0.10 |  |
| *Neutropenia, n (%)* | 4(1.2) | 0(0) | 1 (50) | 4(1.7) | 0(0) | 0.92 |  |
| *Pancytopenia, n (%)* | 1(0.3) | 0(0) | 0 (0) | 1(0.4) | 0(0) | 0.99 |  |
| *Myelodysplastic syndrome, n (%)* | 1(0.3) | 1(1.3) | 0 (0) | 0(0) | 0(0) | 1.00 |  |
| *HLH, n (%)* | 3(0.9) | 3(3.8) | 0 (0) | 0(0) | 0(0) | 1.00 |  |
| *Internal jugular venous thrombosis, n (%)* | 1(0.3) | 0(0) | 0 (0) | 1(0.4) | 0(0) | 0.99 |  |
| Lymphadenopathy, n (%) | 38(11.2) | 8(10.3) | 0 (0) | 20(8.7) | 10(34.5) | <0.001* |  |
| *Hepatomegaly, n (%)* | 28(8.2) | 7(9) | 0 (0) | 19(8.2) | 2(6.9) | 1.00 |  |
| *Splenomegaly, n (%)* | 27(7.9) | 9(11.5) | 0 (0) | 14(6.1) | 4(13.8) | 0.50 |  |
| Hematological cancers, n (%) | 13 (3.8) | 2(2.6) | 0 (0) | 11 (4.7) | 1(3.4) | 0.99 |  |
| *Adult acute lymphoblastic leukemia, n (%)* | 3(0.9) | 0(0) | 0 (0) | 4(1.7) | 0(0) | 0.92 |  |
| *Acute myeloid leukemia, n (%)* | 1(0.3) | 0(0) | 0 (0) | 1(0.4) | 0(0) | 0.99 |  |
| *Lymphoma, n (%)* | 9(2.6) | 2(2.6) | 0 (0) | 6(2.6) | 1(3.4) | 1.00 |  |
| *Hodgkin lymphoma, n (%)* | 3(0.9) | 0(0) | 0 (0) | 3(1.3) | 0(0) | 0.94 |  |
| *Non- Hodgkin lymphoma NHL, n (%)* | 6(1.8) | 1(1.3) | 0 (0) | 4(1.7) | 1(3.4) | 0.94 |  |
| *Diffuse large B-cell lymphoma, n (%)* | 1(0.3) | 1(1.3) | 0 (0) | 0(0) | 0(0) | 1.00 |  |
| *Burkit lymphoma, n (%)* | 1(0.3) | 0(0) | 0 (0) | 1(0.4) | 0(0) | 0.99 |  |
| *Unspecified NHL, n (%)* | 4(1.2) | 0(0) | 0 (0) | 3(1.3) | 1(3.4) | 0.85 |  |
| Solid tumors, n (%) | 9(2.6) | 0(0) | 0(0) | 7 (3.0) | 2(6.9) | 0.76 |  |
| *Osteosarcoma, n (%)* | 1(0.3) | 0(0) | 0 (0) | 1(0.4) | 0(0) | 0.99 |  |
| *Gastrointestinal stromal tumor,n (%)* | 1(0.3) | 0(0) | 0 (0) | 1(0.4) | 0(0) | 0.99 |  |
| *Squamous cell carcinoma, n (%)* | 1(0.3) | 0(0) | 0 (0) | 1(0.4) | 0(0) | 0.99 |  |
| *EBV+ smooth muscle tumor, n (%), n (%)* | 2(0.6) | 0(0) | 0 (0) | 0(0) | 2(6.9) | <0.001* |  |
| *Kaposi’s sarcoma, n (%)* | 1(0.3) | 0(0) | 0 (0) | 1(0.4) | 0(0) | 0.99 |  |
| *Nasal cavity tumor, n (%)* | 1(0.3) | 0(0) | 0 (0) | 1(0.4) | 0(0) | 0.99 |  |
| *Thyroid carcinoma, n (%)* | 1(0.3) | 0(0) | 0 (0) | 1(0.4) | 0(0) | 0.99 |  |
| *Undetermined solid malignancy, n (%)* | 1(0.3) | 0(0) | 0 (0) | 1(0.4) | 0(0) | 0.99 |  |
| ** A comparison between CDC42 and RAC2 groups performed and p-value<0.05 is considered as significant.* | | | | | | | |

**Table S6**: **Details of autoimmune complications in 503 patients with actin-related inborn errors of immunity.**

| **Parameters** | **Total**  **(n=340)** | **Unsolved**  **(n=78)** | **Other actinopathies**  **(n=2)** | **CDC42 pathway**  **(n=231)** | **RAC2 pathway**  **(n=29)** | ***P*-value** |  |
| --- | --- | --- | --- | --- | --- | --- | --- |
| Characterized autoimmunity, n (%) | 90(26.5) | 27(34.6) | 0 (0) | 54(23.4) | 9(31) | 0.84 |  |
| Autoimmune cytopenia, n (%) | 22(6.5) | 3(3.8) | 0 (0) | 17(7.4) | 2(6.9) | 1.00 |  |
| *Evans syndrome, n (%)* | 3(0.9) | 1(1.3) | 0 (0) | 0(0) | 2(6.9) | <0.001* |  |
| *Autoimmune hemolytic anemia, AIHA, n (%)* | 18(5.3) | 3(3.8) | 0 (0) | 13(5.6) | 2(6.9) | 0.99 |  |
| *Immune thrombocytopenic purpura, ITP, n (%)* | 7(2.1) | 1(1.3) | 0 (0) | 5(2.2) | 1(3.4) | 0.98 |  |
| *Plasma cell cheilitis, n (%)* | 1(0.3) | 0(0) | 0 (0) | 1(0.4) | 0(0) | 0.99 |  |
| Inflammatory bowel disease, IBD, n (%) | 12(3.5) | 1(1.3) | 0 (0) | 7(3) | 4(13.8) | 0.06 |  |
| *Early onset IBD, n (%)* | 3(0.9) | 3(3.8) | 0 (0) | 0(0) | 0(0) | 1.00 |  |
| *Other autoimmune enteropathies, n (%)* | 3(0.9) | 0(0) | 0 (0) | 3(1.3) | 0(0) | 0.94 |  |
| *Anticardiolipin syndrome, n (%)* | 1(0.3) | 0(0) | 0 (0) | 1(0.4) | 0(0) | 0.99 |  |
| Celiac, n (%) | 3(0.9) | 0(0) | 0 (0) | 3(1.3) | 0(0) | 0.94 |  |
| Sclerosing cholangitis, n (%) | 4(1.2) | 0(0) | 0 (0) | 4(1.7) | 0(0) | 0.92 |  |
| Autoimmune hepatitis, n (%) | 2(0.6) | 0(0) | 0 (0) | 2(0.9) | 0(0) | 0.97 |  |
| Autoimmune Pericarditis, n (%) | 1(0.3) | 0(0) | 0 (0) | 1(0.4) | 0(0) | 0.99 |  |
| Autoimmune arthritis, n (%) | 3(0.9) | 3(3.8) | 0 (0) | 0(0) | 0(0) | 1.00 |  |
| *Systemic lupus erythematosus, SLE, n (%)* | 1(0.3) | 0(0) | 0 (0) | 1(0.4) | 0(0) | 0.99 |  |
| *Antiphospholipid antibody syndrome, n (%)* | 1(0.3) | 0(0) | 0 (0) | 1(0.4) | 0(0) | 0.99 |  |
| *Autoimmune myopathy, n (%)* | 1(0.3) | 0(0) | 0 (0) | 1(0.4) | 0(0) | 0.99 |  |
| *Guillain-Barré syndrome, n (%)* | 1(0.3) | 1(1.3) | 0 (0) | 0(0) | 0(0) | 1.00 |  |
| *Acute disseminated encephalomyelitis, n (%)* | 1(0.3) | 0(0) | 0 (0) | 1(0.4) | 0(0) | 0.99 |  |
| Vasculitis, n (%) | 15(4.4) | 2(2.6) | 0 (0) | 11(4.8) | 2(6.9) | 0.97 |  |
| *Henoch Schoenlein purpura, n (%)* | 3(0.9) | 0(0) | 0 (0) | 4(1.7) | 0(0) | 0.92 |  |
| *CNS vasculitis, n (%)* | 3(0.9) | 0(0) | 0 (0) | 2(0.9) | 2(6.9) | 0.10 |  |
| *Leukocytoclastic vasculitis, n (%)* | 1(0.3) | 0(0) | 0 (0) | 1(0.4) | 0(0) | 0.99 |  |
| *Lower extremity vasculitis, n (%)* | 2(0.6) | 2(2.6) | 0 (0) | 0(0) | 0(0) | 1.00 |  |
| *Aneurysm, n (%)* | 7(2.1) | 5(6.4) | 0 (0) | 0(0) | 2(6.9) | <0.001* |  |
| Autoimmune Uveitis, n (%) | 1(0.3) | 0(0) | 0 (0) | 1(0.4) | 0(0) | 0.99 |  |
| Autoimmune Retinitis, n (%) | 3(0.9) | 0(0) | 0 (0) | 3(1.3) | 0(0) | 0.94 |  |
| Autoimmune chorioretinitis, n (%) | 1(0.3) | 0(0) | 0 (0) | 1(0.4) | 0(0) | 0.99 |  |
| Nephrotic syndrome, n (%) | 1(0.3) | 0(0) | 0 (0) | 1(0.4) | 0(0) | 0.99 |  |
| Amyloidosis, n (%) | 1(0.3) | 0(0) | 0 (0) | 1(0.4) | 0(0) | 0.99 |  |
| IgA nephropathy, n (%) | 3(0.9) | 1(1.3) | 0 (0) | 2(0.9) | 0(0) | 0.97 |  |
| Diabetes mellitus type 1, n (%) | 1(0.3) | 0(0) | 0 (0) | 1(0.4) | 0(0) | 0.99 |  |
| Autoimmune thyroiditis, n (%) | 3(0.9) | 0(0) | 0 (0) | 3(1.3) | 0(0) | 0.94 |  |
| Psoriasis, n (%) | 2(0.6) | 0(0) | 0 (0) | 0(0) | 2(6.9) | <0.001* |  |
| Vitiligo, n (%) | 1(0.3) | 0(0) | 0 (0) | 0(0) | 1(3.4) | 0.05 |  |
| Alopecia areata, n (%) | 4(1.2) | 0(0) | 0 (0) | 3(1.3) | 1(3.4) | 0.85 |  |
| Auto Anti Positive, n (%) | 6(1.8) | 0(0) | 0 (0) | 3(1.3) | 3(10.3) | 0.02* |  |
| *Anti-Thyroid Peroxidase, Anti-TPO+, n (%)* | 1(0.3) | 0(0) | 0 (0) | 0(0) | 1(3.4) | 0.05 |  |
| *F-actin positive, n (%)* | 1(0.3) | 1(1.3) | 0 (0) | 0(0) | 0(0) | 1.00 |  |
| *Antineutrophil Cytoplasmic Antibodies, ANCA +, n (%)* | 2(0.6) | 0(0) | 0 (0) | 2(0.9) | 0(0) | 0.97 |  |
| *Anti-platelet antibody, n (%)* | 1(0.3) | 0(0) | 0 (0) | 1(0.4) | 0(0) | 0.99 |  |
| *Anti-nuclear antibodies, ANA +, n (%)* | 2(0.6) | 1(1.3) | 0 (0) | 1(0.4) | 0(0) | 0.99 |  |
| *Anti interferon α, n (%)* | 2(0.6) | 0(0) | 0 (0) | 0(0) | 2(6.9) | <0.001* |  |
| *Anti interferon β, n (%)* | 2(0.6) | 0(0) | 0 (0) | 0(0) | 2(6.9) | <0.001* |  |
| *Anti interferon γ, n (%)* | 2(0.6) | 0(0) | 0 (0) | 0(0) | 2(6.9) | <0.001* |  |
| ** A comparison between CDC42 and RAC2 groups performed and p-value<0.05 is considered as significant.* | | | | | | | |

| **TableS7. Clinical and immunologic characteristics of patients with genetic defects in the most common genes associated with actin-related inborn errors of immunity in the MENA region. Only genes with more than 10 patients were presented.** | | | | | | | |
| --- | --- | --- | --- | --- | --- | --- | --- |
| **Parameters** | **CDC42 pathway** | | | | **RAC2 pathway** | | |
|  | ***DOCK8*** | ***WAS*** | ***ARPC1B*** | ***CARMIL2*** | | ***STK4*** | ***DOCK2*** |
| Participants (% of 394 molecularly defined patients) | 212 (53.8) | 97 (24.6) | 12 (3.0) | 17 (4.3) | | 13 (3.2) | 11 (2.7) |
| Age at onset, months, median (IQR) | 6 (3-12) | 1.5 (1-5)^#^ | 4.5 (2-86) | 36 (3-96)^#$^ | | 9 (2-21) | 2 (1-3)^$^ |
| Age at diagnosis, months, median (IQR) | 46 (18-84) | 10 (3-60)^##^ | 91 (84-108) | 110 (48-204) | | 166 (137-198)^##$$^ | 4 (3.5-5.5)^$$^ |
| Age at last visit, months, median (IQR) | 118 (64-170) | 90 (44-174) | 165 (88-188) | 130 (74-236) | | 240 (216-198) | 63 (16-68) |
| Consanguinity, n (%) | 209 (98.5) | 32 (32.9)* | 12 (100) | 17 (100) | | 13 (100) | 11 (100) |
| Male/female | 111/101 | 97/0* | 4/8 | 7/10 | | 5/8 | 7/4 |
| Infections presentations, n (%) | 195(91.9)^@^ | 90(92.7)^##^ | 12(100) ^ω^ | 14(82.3) | | 7(53.8)^##@ ω^ | 9(81.8) |
| Lower respiratory infections, n (%) | 118(55.6)^@@^ | 51(52.5)^#^ | 5(41.6) | 4(23.5)^#@@^ | | 6(46.1) | 6(54.5) |
| Dermatologic infections, n (%) | 149(70.2)^δ^ | 20(20.6) | 6(50.0) | 7(41.1) | | 3(23.0) | 1(9.0) |
| Gastrointestinal infections, n (%) | 44(20.7) | 17(17.5) | 3(25.0) | 5(29.4) | | 2(15.3) | 2(18.1) |
| Genitourinary infections, n (%) | 12(5.6) | 6(6.1) | 0(0) | 0(0) | | 0(0) | 0(0) |
| Nervous system infections, n (%) | 5(2.3) | 10(10.3) | 1(8.3) | 0(0) | | 1(7.6) | 1(9.0) |
| Ophthalmic infections, n (%) | 8(3.7) | 1(1.0) | 1(8.3) | 2(11.7) | | 1(7.6) | 0(0) |
| Musculoskeletal infections, n (%) | 4(1.8) | 5(5.1) | 1(8.3) | 0(0) | | 0(0) | 0(0) |
| Systemic infections, n (%) | 40(18.8) | 17(17.5) | 3(25.0) | 1(5.8) | | 1(7.6) | 1(9.0) |
| Autoimmune presentations, n (%) | 23(10.8) | 50(51.5) | 1(8.3) | 4(23.5) | | 1(7.6) | 1(9.0) |
| Allergic and atopic presentations, n (%) | 201(94.8) ^δ^ | 33 (34.0) | 8(66.6) | 9(52.9) | | 6(46.1) | 5(45.4) |
| Lymphoproliferative presentations, n (%) | 31(14.6) | 17(17.5) | 2(16.6) | 3(17.6) | | 10(76.9)^α^ | 1(9.0) |
| Hematological presentations, n (%) | 12(5.6) | 97 (100)** | 11 (91.6)^β^ | 7(41.1) | | 4(30.7) | 1(9.0) |
| Malignant/oncologic presentations, n (%) | 17(8.0) | 3(3.1) | 0(0) | 1(5.8) | | 3(23.0) ^α^ | 0(0) |
| Absolute leukocytes, cell/uL, median (IQR) | 12650  (9900-16700) | 9000  (7600-11700) | 14725  (8997-17835) | 10700  (8575-11890) | | 2790 ^α^  (2700-3120) | 11300  (10300-12500) |
| Absolute lymphocytes, cell/uL, median (IQR) | 2700  (1742-4252) | 3300  (2000-4670) | 3035  (1446-7300) | 4010  (3370-5625) | | 780 ^α^  (700-910) | 3350  (2100-4200) |
| Absolute neutrophil count, cells /µL, median (IQR) | 5580  (4275-7600) | 4100  (2750-5890) | 7894  (5588-11733) | 4650  (3743-5772) | | 1500  (1270-1800) | 6500  (4450-7120) |
| Absolute eosinophil count, cells /µL, median (IQR) | 3300  (850-7500) ^δ^ | 450  (100-850) | 650  (270-1500) | 150  (80-452) | | 192  (23-850) | 125  (17-495) |
| Platelets, cells /µL, median (IQR) | 405000  (307000-496500) | 29000  (16500-43500)** | 101500  (64500-122000) ^β^ | 319000  (255750-355750) | | 302000  (298000-309000) | 453000  (213000-540000) |
| Mean platelet volume, fl (IQR) | 8.6(7.7-9.3) | 7.1(5.35-8.6)** | 8.4(7.8-8.82) ^β^ | 9.8(8.17-10.3) | | 9.6(8.12-10.9) | 9.5(9.25-9.75) |
| Hemoglobin, g/dL, median (IQR) | 11.2(10.2-12) | 10.1(8.7-11.5)** | 10.8(10.6-12.17) ^β^ | 11.2(10.3-11.9) | | 13(13-13.2) | 11.1(10.5-12.3) |
| Absolute T cell count, cells /µL, median (IQR) | 1155(718-1947) | 2043(1091-3006) | 846 (703-1653)^Ƞ^ | 3074(2196-4278) | | 480(400-586) ^Ƞ^ | 586(435-2100) ^Ƞ^ |
| Absolute T helper count, cells /µL, median (IQR) | 454(270-720) ^ε@@^ | 1038(611-2153) | 565(328-1226) ^Ƞ^ | 1676(1312-2506) | | 223(132-226) ^Ƞ^ | 325(176-352) ^Ƞ^ |
| Absolute T cytotoxic, cells /µL, median (IQR) | 553(226-903) | 589(327-920) | 312(264-339) ^Ƞ^ | 1328(807-1631) | | 310(270-315) ^Ƞ^ | 333(207-1400) ^Ƞ^ |
| Absolute B cell count, cells /µL, median (IQR) | 661(260-1208) | 360(172-713) | 167(534-2463) | 635(226-1105) | | 75(60-102) ^α^ | 693(316-1728) |
| Absolute NK cell count, cells /µL, median (IQR) | 207(45-381) | 494(251-798) | 153(90-624) | 105(64-186) | | 956(30-110) | 74(37-104) ^κ κ^ |
| IgG, mg/dL, median (IQR) | 1245(944-1522) ^Ɣ@ε^ | 604(443-1178)^λε^ | 1205(1170-1367)^λμ^ | 925.5(608-1126) | | 595(462-765)^@μ^ | 539(350-785)^Ɣ^ |
| IgA, mg/dL, median (IQR) | 135(61-213) | 122(56-241) | 432(302-1646) | 187(118-306) | | 52(27-86) | 60(22-131) |
| IgM, mg/dL, median (IQR) | 44(24-83) | 24(11-86)^***^ | 88(54-230) | 124(98-196) | | 74(41-95) | 28(23-54) ^κ^ |
| IgE, IU/ml, median (IQR) | 2000(631-5000) ^δ^ | 77(23-408) | 146.1(26-516) | 20(7-136) | | 141(58-146) | 32(27-131) |
| ** Significant between WAS and all other gene groups. ** Significant between WAS and all other gene groups except ARPC1B. *** Significant between WAS and all other gene groups except DOCK2. ^ε^ Significant between WAS and DOCK8 groups. ^#^ Significant between WAS and CARMIL2 groups. ^##^ Significant between WAS and STK4 groups. ^λ^ Significant between WAS and ARPC1B groups.*  *^δ^ Significant between DOCK8 and all other gene groups. ^@^  Significant between DOCK8 and STK4 groups. ^@@^ Significant between DOCK8 and CARMIL2 groups. . ^Ɣ^ Significant between DOCK8 and DOCK2 groups.*  *^κ^ Significant between DOCK2 and all other gene groups except WAS.  ^κ κ^ Significant between DOCK2 and all other gene groups except CARMIL2. ^$^Significant between DOCK2 and CARMIL2 groups. ^$$^ Significant between DOCK2 and STK4 groups. ^Ƞ^  Significant lower in DOCK2,STK4 and ARPIC1B compared to WAS and CARMIL2 groups.*  *^α^ Significant between STK4 and all other gene groups. ^ω^ Significant between STK4 and ARPC1B groups. ^μ^ Significant between STK4 and ARPC1B groups.*  *^β^ Significant between ARPC1B and all other gene groups except WAS.* | | | | | | | |

**TableS8.** List of identified pathogenic/likely pathogenic variants and their demographic and clinical presentations.

| Gene | Mutation | Type of mutation | Zygosity | Gender | Age of Onset (months) | Age of Diagnosis (months) | Severe infection | Autoimmunity | Atopy | Other hematologic | Lymphoproliferation | Cancer |
| --- | --- | --- | --- | --- | --- | --- | --- | --- | --- | --- | --- | --- |
| *ARPC1B* | c.-14+1G>A | SP | Hom | F | 1 | 114 | Yes |  | Yes | Yes |  |  |
| *ARPC1B* | c.-14+1G>A | SP | Hom | M | 3 | 6 | Yes |  |  | Yes |  |  |
| *ARPC1B* | c.392+1G>C | SP | Hom | F | 4.5 | 6 | Yes |  |  |  |  |  |
| *ARPC1B* | p.D130GfsX63 | FS | Hom | F | 72 | 132 | Yes |  |  | Yes |  |  |
| *ARPC1B* | p.E300PfsX153 | FS | Hom | F | 92 | 93 |  |  | Yes |  |  |  |
| *ARPC1B* | p.E300PfsX153 | FS | Hom | M | 91 | 91 |  |  | Yes |  |  |  |
| *ARPC1B* | p.E300PfsX153 | FS | Hom | F | 86 | 89 |  |  | Yes |  |  |  |
| *ARPC1B* | p.E300PfsX153 | FS | Hom | F | 40 | 76 |  |  | Yes |  |  |  |
| *ARPC1B* | p.E300PfsX153 | FS | Hom | F | 45 | 70 |  |  | Yes |  |  |  |
| *ARPC1B* | p.E300PfsX153 | FS | Hom | M | 43 | 82 |  |  | Yes |  |  |  |
| *ARPC1B* | p.E300PfsX153 | FS | Hom | M | 2 | 108 | Yes |  |  |  |  |  |
| *ARPC1B* | p.I67T | MS | Hom | F | 1 | 84 | Yes |  |  | Yes |  |  |
| *CARMIL2* | c.1920-7G>A | SP | Hom | F | 120 | 252 | Yes |  |  | Yes |  |  |
| *CARMIL2* | c.1920-7G>A | SP | Hom | F | 96 | 204 | Yes |  |  | Yes |  |  |
| *CARMIL2* | c.467_537del | LD | Hom | M | 2 | 48 |  |  |  |  | Yes |  |
| *CARMIL2* | p.C155VfsX54 | FS | Hom | F | 108 | 228 | Yes |  |  |  |  |  |
| *CARMIL2* | p.C155VfsX54 | FS | Hom | F | 48 | 140 | Yes |  |  |  |  |  |
| *CARMIL2* | p.D615Y | MS | Hom | M | 1 | 66 | Yes |  |  | Yes |  |  |
| *CARMIL2* | p.L525Q | MS | Hom | M | 120 | 264 | Yes |  |  |  |  |  |
| *CARMIL2* | p.Q389X | ST | Hom | M | 4 | 19 | Yes |  |  |  |  |  |
| *CARMIL2* | p.R301LfsX6 | FS | Hom | F | 30 | 180 | Yes |  | Yes |  |  |  |
| *CARMIL2* | p.R385T | MS | Hom | M | 3 | 110 |  |  | Yes |  | Yes | Yes |
| *CARMIL2* | p.R385T | MS | Hom | M | 36 | 48 | Yes |  | Yes |  |  |  |
| *CARMIL2* | p.R385T | MS | Hom | F | 3 | 60 | Yes |  | Yes |  |  |  |
| *CARMIL2* | p.R385T | MS | Hom | F | 4 | 132 | Yes |  |  |  |  |  |
| *CARMIL2* | p.R385T | MS | Hom | M | 3 | 104 | Yes |  |  | Yes | Yes | Yes |
| *CARMIL2* | p.R385T | MS | Hom | F | 44 | 46 |  |  |  |  |  |  |
| *CARMIL2* | p.R385T | MS | Hom | F | 44 | 46 | Yes |  |  |  |  |  |
| *CARMIL2* | p.R646P | MS | Hom | F | 216 | 240 | Yes |  |  |  |  |  |
| *CEBPE* | p.P75LfsX35 | FS | Hom | M | 1 | 168 | Yes |  |  |  | Yes |  |
| *CORO1A* | p.R354X | ST | Hom | F | 15 | 72 | Yes | Yes |  |  |  |  |
| *CORO1A* | p.R354X | ST | Hom | F | 35 | 36 |  |  |  |  |  |  |
| *CORO1A* | p.R245W | MS | Hom | M | 28 | 30 | Yes |  |  |  | Yes |  |
| *CORO1A* | p.R245W | MS | Hom | F | 30 | 120 | Yes |  |  |  |  |  |
| *CORO1A* | p.R245W | MS | Hom | M | 36 | 156 | Yes |  |  |  |  |  |
| *CORO1A* | p.G413R | MS | Hom | F | 13 | 13 | Yes |  |  |  |  |  |
| *CORO1A* | p.R245W | MS | Hom | F | 6 | 156 | Yes |  |  |  |  |  |
| *CORO1A* | p.R245W | MS | Hom | M | 11 | 5 |  |  |  |  |  |  |
| *DOCK2* | c.902–1078del | LD | Hom | F | 1 | 4 | Yes |  | Yes |  |  |  |
| *DOCK2* | p.F744CfsX27 | FS | Hemi | M | 1 | 12 | Yes |  | Yes |  |  |  |
| *DOCK2* | p.I505SfsX28 | FS | Hemi | F | 1 | 5 |  |  | Yes |  |  |  |
| *DOCK2* | p.R1104W | MS | Hemi | F | 2 | 4 |  |  | Yes |  |  |  |
| *DOCK2* | p.R508X | ST | Hemi | M | 1 | 3 | Yes |  |  |  |  |  |
| *DOCK2* | p.R508X | ST | Hemi | F | 3 | 3 | Yes |  |  |  |  |  |
| *DOCK2* | p.R751S | MS | Hemi | M | 3 | 7 |  |  |  |  |  |  |
| *DOCK2* | p.R751S | MS | Hemi | M | 6 | 6 | Yes |  |  |  |  |  |
| *DOCK2* | p.W623X | ST | Hom | M | 0 | 1 |  | Yes |  |  |  |  |
| *DOCK2* | p.W623X | ST | Hom | M | 3 | 4 | Yes |  |  |  |  |  |
| *DOCK2* | p.Y1242YfsX33 | FS | Hemi | M | 3 | 5 | Yes |  |  |  |  |  |
| *DOCK8* | c.1803+1G>C | SP | Hom | M | 12 | 56 |  |  | Yes |  |  |  |
| *DOCK8* | c.1869-1G>C | SP | Hom | M | 23 | 29 | Yes |  | Yes |  |  |  |
| *DOCK8* | c.1869-1G>C | SP | Hom | F | 6 | 6 | Yes |  | Yes |  | Yes |  |
| *DOCK8* | c.1869-1G>C | SP | Hom | F | 36 | 60 | Yes |  | Yes |  |  |  |
| *DOCK8* | c.1869-1G>C | SP | Hom | M | 24 | 70 |  |  | Yes |  |  |  |
| *DOCK8* | c.1869-1G>C | SP | Hom | F | 24 | 70 |  |  | Yes |  |  |  |
| *DOCK8* | c.1869-1G>C | SP | Hom | F | 13 | 15 | Yes |  | Yes |  |  |  |
| *DOCK8* | c.1869-1G>C | SP | Hom | M | 12 | 24 | Yes |  | Yes |  |  |  |
| *DOCK8* | c.1869-1G>C | SP | Hom | M | 6 | 6 | Yes |  | Yes |  |  |  |
| *DOCK8* | c.1914-7insT | FS | Hom | F | 1.5 | 2 |  |  | Yes |  |  |  |
| *DOCK8* | c.2007+1G>C | SP | Hom | F | 10 | 53 |  |  | Yes |  |  |  |
| *DOCK8* | c.2206-2C>G | SP | Hom | M | 6 | 60 | Yes |  |  |  |  |  |
| *DOCK8* | c.2206-3C>G | SP | Hom | F | 6 | 55 | Yes |  | Yes |  |  |  |
| *DOCK8* | c.2555-1G>A | SP | Hom | M | 13 | 57 |  |  | Yes |  |  |  |
| *DOCK8* | c.2626-3C>T/p.H974N | SP +MS | Comp het | F | 36 | 300 | Yes |  | Yes |  |  | Yes |
| *DOCK8* | c.4241+1G>T | SP | Hom | F | 14 | 59 |  |  | Yes |  |  |  |
| *DOCK8* | c.4626+76A>C | SP | Hom | F | 6 | 33 | Yes |  |  |  |  |  |
| *DOCK8* | c.4682+1G>T | SP | Hom | F | 3 | 10 | Yes |  | Yes |  | Yes |  |
| *DOCK8* | c.4886+1G>T | SP | Hom | F | 8 | 50 |  |  | Yes |  |  |  |
| *DOCK8* | c.741+5G>T | SP | Hom | M | 9 | 51 |  |  | Yes |  |  |  |
| *DOCK8* | c.741+5G>T | SP | Hom | F | 12 | 55 |  |  | Yes |  |  |  |
| *DOCK8* | c.742-21C>A | SP | Hom | M | 1 | 65 | Yes |  | Yes |  |  |  |
| *DOCK8* | EX1-10del | LD | Hom | F | 3 | 4 | Yes |  | Yes |  | Yes |  |
| *DOCK8* | EX11-13del | LD | Hom | M | 7 | 50 |  |  | Yes |  |  |  |
| *DOCK8* | EX11-14del | LD | Hom | M | 15 | 59 |  |  | Yes |  |  |  |
| *DOCK8* | EX1-11del | LD | Hom | F | 2 | 70 | Yes |  |  |  |  |  |
| *DOCK8* | EX1-11del | LD | Hom | F | 1 | 122 | Yes |  | Yes |  |  |  |
| *DOCK8* | EX1-11del | LD | Hom | F | 3 | 57 | Yes |  | Yes |  |  |  |
| *DOCK8* | EX1-11del | LD | Hom | M | 11 | 19 | Yes |  | Yes |  |  |  |
| *DOCK8* | EX1-11del | LD | Hom | M | 60 | 120 | Yes |  | Yes |  |  |  |
| *DOCK8* | EX1-11del | LD | Hom | M | 30 | 36 | Yes | Yes |  |  |  |  |
| *DOCK8* | EX11-28del | LD | Hom | M | 12 | 54 |  |  | Yes |  |  |  |
| *DOCK8* | EX1-14del | LD | Hom | M | 6 | 90 | Yes |  | Yes |  |  |  |
| *DOCK8* | EX1-19del | LD | Hom | M | 30 | 168 | Yes |  | Yes |  |  |  |
| *DOCK8* | EX1-19del | LD | Hom | M | 5 | 102 | Yes |  |  |  |  |  |
| *DOCK8* | EX1-19del | LD | Hom | M | 0 | 2 | Yes |  |  |  |  |  |
| *DOCK8* | EX1-19del | LD | Hom | M | 24 | 170 | Yes |  | Yes |  |  |  |
| *DOCK8* | EX1-19del | LD | Hom | M | 12 | 120 | Yes |  | Yes |  |  |  |
| *DOCK8* | EX1-19del | LD | Hom | F | 12 | 18 | Yes |  |  |  |  |  |
| *DOCK8* | EX1-20del | LD | Hom | M | 36 | 84 | Yes |  |  |  |  |  |
| *DOCK8* | EX1-22del | LD | Hom | M | 10 | 60 | Yes |  | Yes |  |  |  |
| *DOCK8* | EX1-24del | LD | Hom | F | 1 | 7 |  |  | Yes |  |  |  |
| *DOCK8* | EX1-25del | LD | Hom | M | 6 | 94 | Yes |  | Yes |  |  |  |
| *DOCK8* | EX1-26del | LD | Hom | M | 48 | 195 | Yes |  |  |  |  |  |
| *DOCK8* | EX1-26del | LD | Hom | F | 6 | 37 |  |  | Yes |  |  |  |
| *DOCK8* | EX1-26del | LD | Hom | M | 4 | 6 | Yes |  | Yes |  |  |  |
| *DOCK8* | EX1-26del | LD | Hom | F | 6 | 37 | Yes |  | Yes |  |  |  |
| *DOCK8* | EX1-26del | LD | Hom | M | 1 | 12 | Yes |  | Yes |  |  |  |
| *DOCK8* | EX1-26del | LD | Hom | F | 4 | 60 | Yes |  | Yes |  |  |  |
| *DOCK8* | EX1-26del | LD | Hom | M | 12 | 50 |  |  | Yes |  |  |  |
| *DOCK8* | EX1-26del | LD | Hom | M | 6 | 48 |  |  | Yes |  |  |  |
| *DOCK8* | EX1-26del | LD | Hom | M | 12 | 96 | Yes |  | Yes |  |  |  |
| *DOCK8* | EX1-26del | LD | Hom | M | 6 | 90 |  |  | Yes |  |  |  |
| *DOCK8* | EX1-26del | LD | Hom | M | 12 | 96 | Yes |  | Yes |  |  |  |
| *DOCK8* | EX1-26del | LD | Hom | M | 30 | 36 |  |  | Yes |  |  |  |
| *DOCK8* | EX1-27del | LD | Hom | M | 8 | 59 | Yes |  | Yes |  |  |  |
| *DOCK8* | EX1-27del | LD | Hom | M | 6 | 80 | Yes |  | Yes |  |  |  |
| *DOCK8* | EX1-27del | LD | Hom | F | 18 | 50 | Yes |  | Yes |  |  |  |
| *DOCK8* | EX1-28del | LD | Hom | M | 2 | 6 |  |  | Yes |  |  |  |
| *DOCK8* | EX1-2del | LD | Hom | M | 6 | 54 | Yes |  | Yes |  |  |  |
| *DOCK8* | EX1-2del | LD | Hom | M | 3 | 10 | Yes |  | Yes |  |  |  |
| *DOCK8* | EX1-2del | LD | Hom | F | 4 | 14 | Yes |  | Yes |  |  |  |
| *DOCK8* | EX1-2del | LD | Hom | F | 8 | 70 | Yes |  | Yes |  |  |  |
| *DOCK8* | EX1-2del | LD | Hom | M | 1 | 4 |  |  | Yes |  |  |  |
| *DOCK8* | EX1-2del | LD | Hom | M | 6 | 56 | Yes |  | Yes |  |  |  |
| *DOCK8* | EX1-2del | LD | Hom | F | 3 | 32 | Yes |  | Yes |  |  |  |
| *DOCK8* | EX1-37del | LD | Hom | F | 7 | 76 | Yes |  |  |  |  |  |
| *DOCK8* | EX1-37del | LD | Hom | M | 6 | 50 | Yes |  |  |  |  |  |
| *DOCK8* | EX1-37del | LD | Hom | F | 1 | 48 | Yes |  |  |  |  |  |
| *DOCK8* | EX1-37del | LD | Hom | M | 23 | 26 | Yes |  |  |  |  |  |
| *DOCK8* | EX1-37del | LD | Hom | M | 23 | 26 | Yes |  |  |  |  |  |
| *DOCK8* | EX1-37del | LD | Hom | M | 24 | 36 | Yes |  | Yes |  |  |  |
| *DOCK8* | EX1-3del | LD | Hom | F | 11 | 59 |  |  | Yes |  |  |  |
| *DOCK8* | EX1-42del | LD | Hom | M | 8 | 96 | Yes |  |  |  |  |  |
| *DOCK8* | EX1-44del | LD | Hom | F | 12 | 52 |  |  | Yes |  |  |  |
| *DOCK8* | EX1-44del | LD | Hom | F | 18 | 84 | Yes |  | Yes |  |  |  |
| *DOCK8* | EX1-44del | LD | Hom | F | 48 | 78 | Yes |  | Yes |  |  |  |
| *DOCK8* | EX1-44del | LD | Hom | F | 12 | 36 | Yes |  | Yes | Yes |  |  |
| *DOCK8* | EX1-48del | LD | Hom | F | 11 | 48 |  |  | Yes |  |  |  |
| *DOCK8* | EX1-48del | LD | Hom | M | 30 | 119 | Yes |  |  |  |  |  |
| *DOCK8* | EX16-23del | LD | Hom | M | 1 | 150 | Yes |  | Yes |  |  |  |
| *DOCK8* | EX1-7del | LD | Hom | F | 4 | 12 | Yes |  | Yes |  |  |  |
| *DOCK8* | EX18-24del | LD | Hom | F | 11 | 56 |  |  | Yes |  |  |  |
| *DOCK8* | EX1-9del | LD | Hom | F | 12 | 36 | Yes |  |  |  |  |  |
| *DOCK8* | EX1del | LD | Hom | F | 6 | 33 | Yes |  |  |  |  |  |
| *DOCK8* | EX2-11del | LD | Hom | M | 12 | 50 |  |  | Yes |  |  |  |
| *DOCK8* | EX2-12del | LD | Hom | F | 8 | 16 | Yes |  | Yes |  |  |  |
| *DOCK8* | EX2-15del | LD | Hom | M | 10 | 54 |  |  | Yes |  |  |  |
| *DOCK8* | EX2-15del | LD | Hom | M | 6 | 56 |  |  | Yes |  |  |  |
| *DOCK8* | EX2-26del | SP | Hom | M | 3 | 10 | Yes |  | Yes |  |  |  |
| *DOCK8* | EX2-26del | LD | Hom | F | 12 | 58 |  |  | Yes |  |  |  |
| *DOCK8* | EX22del | ST | Hom | M | 12 | 60 | Yes |  | Yes |  |  |  |
| *DOCK8* | EX23-30del | LD | Hom | F | 11 | 62 |  |  | Yes |  |  |  |
| *DOCK8* | EX23-30del | LD | Hom | M | 9 | 51 |  |  | Yes |  |  |  |
| *DOCK8* | EX23del | LD | Hom | F | 12 | 60 | Yes | Yes | Yes |  |  |  |
| *DOCK8* | EX24-48del | LD | Hom | F | 3 | 36 |  |  | Yes |  |  |  |
| *DOCK8* | EX25-26del | LD | Hom | M | 0.8 | 25 | Yes |  | Yes |  |  |  |
| *DOCK8* | EX25-26del | LD | Hom | M | 12 | 59 |  |  | Yes |  |  |  |
| *DOCK8* | EX25-26del | LD | Hom | M | 10 | 56 |  |  | Yes |  |  |  |
| *DOCK8* | EX25-26del | LD | Hom | F | 12 | 49 |  |  | Yes |  |  |  |
| *DOCK8* | EX27-48del | LD | Hom | F | 4 | 90 | Yes |  |  |  |  |  |
| *DOCK8* | EX27del | LD | Hom | M | 4 | 48 | Yes |  | Yes |  |  |  |
| *DOCK8* | EX27del | MS | Hom | F | 12 | 57 | Yes |  | Yes |  |  |  |
| *DOCK8* | EX27del | LD | Hom | F | 6 | 48 | Yes |  |  |  |  |  |
| *DOCK8* | EX27del | LD | Hom | F | 1 | 48 | Yes |  |  |  |  |  |
| *DOCK8* | EX27del | LD | Hom | F | 1 | 9 | Yes |  | Yes |  |  |  |
| *DOCK8* | EX27del | LD | Hom | F | 1 | 36 |  |  | Yes |  |  |  |
| *DOCK8* | EX2-7del | SP | Hom | M | 1 | 180 | Yes |  | Yes |  |  |  |
| *DOCK8* | EX2-7del | SP | Hom | M | 1 | 150 | Yes | Yes | Yes | Yes |  |  |
| *DOCK8* | EX28-44del | LD | Hom | F | 48 | 156 | Yes |  | Yes |  | Yes |  |
| *DOCK8* | EX28-44del | LD | Hom | M | 5 | 48 | Yes |  |  |  | Yes |  |
| *DOCK8* | EX28-44del | LD | Hom | F | 4 | 84 | Yes |  | Yes |  | Yes |  |
| *DOCK8* | EX28-48del | LD | Hom | F | 3 | 48 | Yes |  |  |  |  |  |
| *DOCK8* | EX28-48del | LD | Hom | F | 12 | 24 | Yes |  | Yes |  |  |  |
| *DOCK8* | EX28-49del | LD | Hom | M | 12 | 105 | Yes |  | Yes |  | Yes | Yes |
| *DOCK8* | EX2del | LD | Hom | F | 7 | 12 |  |  | Yes |  |  |  |
| *DOCK8* | EX2del | LD | Hom | F | 8 | 69 |  |  | Yes |  |  |  |
| *DOCK8* | EX2del | LD | Hom | F | 11 | 52 |  |  | Yes |  |  |  |
| *DOCK8* | EX2del | LD | Hom | M | 15 | 65 |  |  | Yes |  |  |  |
| *DOCK8* | EX2del |  | Hom | F | 24 | 23 | Yes |  | Yes |  |  |  |
| *DOCK8* | EX30-49del | LD | Hom | M | 60 | 108 | Yes | Yes | Yes |  |  |  |
| *DOCK8* | EX33del | LD | Hom | M | 9 | 26 |  |  | Yes |  |  |  |
| *DOCK8* | EX3-48del | LD | Hom | M | 6 | 32 |  |  | Yes |  |  |  |
| *DOCK8* | EX37del | LD | Hom | F | 6 | 90 | Yes |  | Yes |  |  |  |
| *DOCK8* | EX4-12del | LD | Hom | M | 11 | 23 |  |  | Yes |  |  |  |
| *DOCK8* | EX4-12del | LD | Hom | M | 6 | 12 |  |  | Yes |  |  |  |
| *DOCK8* | EX41-43del | LD | Hom | M | 12 | 54 |  |  | Yes |  |  |  |
| *DOCK8* | EX45-48del | LD | Hom | M | 72 | 218 | Yes |  | Yes |  | Yes |  |
| *DOCK8* | EX4-5del | LD | Hom | M | 10 | 47 |  |  | Yes |  |  |  |
| *DOCK8* | EX4-5del | LD | Hom | F | 11 | 22 |  |  | Yes |  |  |  |
| *DOCK8* | EX48del | LD | Hom | F | 17 | 23 |  |  | Yes |  |  |  |
| *DOCK8* | EX5-29del | LD | Hom | F | 8 | 44 | Yes |  | Yes |  |  |  |
| *DOCK8* | EX6-15del | LD | Hom | F | 12 | 56 |  |  | Yes |  |  |  |
| *DOCK8* | EX6-15del | LD | Hom | F | 11 | 33 |  |  | Yes |  |  |  |
| *DOCK8* | EX7-11del | LD | Hemi | F | 12 | 36 | Yes |  | Yes |  |  |  |
| *DOCK8* | EX7-11del | LD | Hom | F | 24 | 36 | Yes |  | Yes |  |  |  |
| *DOCK8* | EX7-8del | LD | Hom | F | 0 | 5 | Yes |  | Yes |  |  |  |
| *DOCK8* | EX8-10del | LD | Hom | M | 30 | 36 | Yes |  | Yes |  |  |  |
| *DOCK8* | EX9-12del | LD | Hom | M | 0.7 | 0.7 | Yes |  | Yes |  |  |  |
| *DOCK8* | p.D1064VfsX29 | FS | Hom | F | 4 | 40 | Yes |  | Yes |  | Yes |  |
| *DOCK8* | p.D1064VfsX29 | FS | Hom | M | 6 | 12 | Yes |  | Yes |  |  |  |
| *DOCK8* | p.D85TfsX46 | FS | Hom | F | 6 | 60 | Yes |  | Yes |  |  |  |
| *DOCK8* | p.E1301X | ST | Hom | M | 10 | 37 |  |  | Yes |  |  |  |
| *DOCK8* | p.E1301X | ST | Hom | F | 0.1 | 6 | Yes |  | Yes |  |  |  |
| *DOCK8* | p.E1301X | ST | Hom | F | 0.1 | 1 | Yes |  | Yes |  |  |  |
| *DOCK8* | p.E1301X | ST | Hom | M | 0.1 | 8 | Yes |  | Yes |  |  |  |
| *DOCK8* | p.E1401X | ST | Hom | M | 10 | 53 |  |  | Yes |  |  |  |
| *DOCK8* | p.E1401X | ST | Hom | M | 14 | 23 |  |  | Yes |  |  |  |
| *DOCK8* | p.E475GfsX5 | FS | Hom | F | 11 | 55 |  |  | Yes |  |  |  |
| *DOCK8* | p.E750X | ST | Hom | F | 6 | 22 | Yes |  |  |  |  |  |
| *DOCK8* | p.G1988fsX1990 | FS | Hom | M | 18 | 24 | Yes |  | Yes |  |  |  |
| *DOCK8* | p.G46D | MS | Hom | M | 30 | 42 | Yes |  | Yes |  |  |  |
| *DOCK8* | p.G46D | MS | Hom | M | 2 | 96 | Yes |  |  |  |  |  |
| *DOCK8* | p.G46D | MS | Hom | F | 8 | 11 | Yes |  |  |  |  |  |
| *DOCK8* | p.H1074N | MS | Hom | F | 1.5 | 96 | Yes |  | Yes |  |  |  |
| *DOCK8* | p.H1509Q | MS | Hom | M | 18 | 225 | Yes |  |  |  |  |  |
| *DOCK8* | p.H1772AfsX57 | FS | Hom | M | 1 | 59 |  |  | Yes |  |  |  |
| *DOCK8* | p.H541N | MS | Hom | F | 11 | 52 |  |  | Yes |  |  |  |
| *DOCK8* | p.I1020V | MS | Hom | F | 2 | 1 | Yes |  |  |  |  |  |
| *DOCK8* | p.I1020V | MS | Hom | F | 3 | 3 | Yes |  | Yes |  |  |  |
| *DOCK8* | p.I1020V/p.H541Q | MS | Comp het | M | 12 | 56 |  |  | Yes |  |  |  |
| *DOCK8* | p.K135fsX | FS | Hom | F | 8 | 53 |  |  | Yes |  |  |  |
| *DOCK8* | p.K1886EfsX10 | FS | Hom | M | 17 | 22 |  |  | Yes |  |  |  |
| *DOCK8* | p.K1955del | ID | Hom | M | 11 | 54 |  |  | Yes |  |  |  |
| *DOCK8* | p.K43R | MS | Hom | F | 6 | 24 | Yes |  |  |  |  |  |
| *DOCK8* | p.L1052PfsX7 | FS | Hom | F | 4 | 12 |  |  | Yes |  |  |  |
| *DOCK8* | p.L1060SfsX33 | FS | Hom | F | 3 | 84 | Yes |  | Yes |  |  |  |
| *DOCK8* | p.L1060SfsX33 | FS | Hom | M | 6 | 6 | Yes |  | Yes |  |  |  |
| *DOCK8* | p.L1060SfsX33 | FS | Hom | M | 1 | 18 | Yes |  | Yes |  |  |  |
| *DOCK8* | p.L1060SfsX33 | LD | Hom | M | 12 | 36 | Yes |  | Yes |  |  |  |
| *DOCK8* | p.L1060SfsX33 | LD | Hom | F | 0.1 | 2 | Yes |  | Yes |  |  |  |
| *DOCK8* | p.L1060SfsX33 | LD | Hom | M | 3 | 22 |  |  | Yes |  |  |  |
| *DOCK8* | p.L1193V | FS | Hom | M | 3 | 12 |  |  | Yes |  |  |  |
| *DOCK8* | p.L1193V | MS | Hom | F | 22 | 42 |  |  | Yes |  |  |  |
| *DOCK8* | p.L1330V | MS | Hom | M | 61 | 144 |  |  | Yes |  |  |  |
| *DOCK8* | p.M1114YfsX4 | FS | Hom | F | 2 | 60 | Yes |  | Yes |  |  |  |
| *DOCK8* | p.M1852R | MS | Hom | M | 2 | 30 | Yes |  | Yes |  |  |  |
| *DOCK8* | p.M1852R | MS | Hom | M | 6 | 15 | Yes |  | Yes |  |  |  |
| *DOCK8* | p.N1000KfsX22 | FS | Hom | F | 6 | 93 | Yes |  | Yes | Yes |  |  |
| *DOCK8* | p.Q1078PfsX7 | FS | Hom | F | 12 | 55 |  |  | Yes |  |  |  |
| *DOCK8* | p.Q176H | SP | Hom | M | 6 | 42 | Yes |  | Yes |  |  |  |
| *DOCK8* | p.Q474H | MS | Hom | M | 18 | 108 | Yes | Yes |  |  | Yes | Yes |
| *DOCK8* | p.Q498X | ST | Hom | F | 9 | 9 | Yes |  | Yes |  |  |  |
| *DOCK8* | p.Q498X | ST | Hom | M | 5 | 24 | Yes |  | Yes |  |  |  |
| *DOCK8* | p.Q498X | LD | Hom | F | 3 | 15 | Yes |  |  |  |  |  |
| *DOCK8* | p.Q498X | LD | Hom | F | 1 | 9 | Yes |  | Yes |  |  |  |
| *DOCK8* | p.Q498X | LD | Hom | M | 4 | 36 | Yes |  | Yes |  |  |  |
| *DOCK8* | p.Q498X | LD | Hom | F | 12 | 18 | Yes |  | Yes |  |  |  |
| *DOCK8* | p.R1154C | MS | Hom | M | 6 | 20 | Yes |  | Yes |  | Yes |  |
| *DOCK8* | p.R1370PfsX1395 | FS | Hom | F | 10 | 41 | Yes |  | Yes |  |  |  |
| *DOCK8* | p.R1370PfsX1395 | FS | Hom | M | 22 | 35 | Yes |  | Yes |  |  |  |
| *DOCK8* | p.R18fsX34 | FS | Hom | F | 11 | 58 |  |  | Yes |  |  |  |
| *DOCK8* | p.R18fsX34 | FS | Hom | F | 10 | 39 | Yes |  | Yes |  |  |  |
| *DOCK8* | p.R18fsX34 | FS | Hom | M | 8 | 49 |  |  | Yes |  |  |  |
| *DOCK8* | p.R443KfsX15 | FS | Hom | M | 5 | 22 | Yes |  | Yes |  |  |  |
| *DOCK8* | p.R482X | ST | Hom | M | 1 | 6 | Yes |  | Yes |  | Yes |  |
| *DOCK8* | p.R482X | ST | Hom | F | 0.4 | 10 | Yes |  | Yes |  | Yes | Yes |
| *DOCK8* | p.R908KfsX15 | FS | Hom | F | 12 | 59 |  |  | Yes |  |  |  |
| *DOCK8* | p.S1711X | ST | Hom | M | 2 | 2 | Yes |  | Yes |  |  |  |
| *DOCK8* | p.S4182X | ST | Hom | M | 36 | 84 | Yes |  | Yes |  |  |  |
| *DOCK8* | p.S868X | ST | Hom | F | 12 | 46 | Yes |  | Yes |  |  |  |
| *DOCK8* | p.T1610SfsX11 | FS | Hom | F | 36 | 48 | Yes |  |  |  |  |  |
| *DOCK8* | p.T1610SfsX11 | FS | Hom | M | 72 | 96 | Yes |  |  |  |  |  |
| *DOCK8* | p.T1610SfsX11 | FS | Hom | M |  | 84 | Yes |  | Yes |  |  |  |
| *DOCK8* | p.T1610SfsX11 | FS | Hom | F | 18 | 84 | Yes |  | Yes |  |  |  |
| *DOCK8* | p.T1610SfsX11 | FS | Hom | M | 72 | 102 | Yes |  | Yes |  | Yes | Yes |
| *DOCK8* | p.T1610SfsX11 | FS | Hom | F | 36 | 48 | Yes | Yes | Yes |  |  |  |
| *DOCK8* | p.T1610SfsX11 | FS | Hom | F | 18 | 84 | Yes | Yes | Yes |  | Yes |  |
| *DOCK8* | p.W1544X | ST | Hom | F | 1 | 36 | Yes |  | Yes |  |  |  |
| *DOCK8* | p.Y1634X | ST | Hom | F | 6 | 84 | Yes |  | Yes |  |  |  |
| *DOCK8* | p.Y1634X | ST | Hom | M | 1 | 1 | Yes |  |  |  |  |  |
| *DOCK8* | p.Y1634X | ST | Hom | M | 4 | 18 | Yes |  | Yes |  |  |  |
| *MSN* | p.R171W | MS | Hemi | M | 44 | 136 | Yes |  |  |  |  |  |
| *MSN* | c.796-3 C>G | SP | Hemi | M | 48 | 141 | Yes |  |  |  |  |  |
| *NCKAP1L* | p.M372V | MS | Hom | M | 13 | 32 |  |  |  |  |  |  |
| *NCKAP1L* | p.V141F | MS | Hom | F | 14 | 28 | Yes |  |  |  |  |  |
| *NCKAP1L* | p.V141F | MS | Hom | M | 12 | 34 | Yes |  |  |  |  |  |
| *PSTPIP1* | p.Q354R | MS | Het | M | 7 | 100 | Yes |  |  |  |  |  |
| *PSTPIP1* | p.A39T | MS | Het | M | 9 | 104 | Yes |  |  |  |  |  |
| *PSTPIP1* | p.G258A | MS | Het | M | 5 | 98 | Yes |  |  |  |  |  |
| *PSTPIP1* | p.E257K | MS | Het | F | 6 | 110 | Yes |  |  |  |  |  |
| *PSTPIP1* | p.R275Q / p.D449G | MS | Het | F | 7 | 93 | Yes |  |  |  |  |  |
| *RAC2* | p.E131G | MS | Het | M | 1 | 6 | Yes | Yes |  |  |  |  |
| *RAC2* | p.Trp56X | ST | Hom | M | 2 | 10 | Yes | Yes |  |  |  |  |
| *RAC2* | p.Trp56X | ST | Hom | F | 1 | 11 | Yes | Yes |  |  |  |  |
| *RAC2* | p.Trp56X | ST | Hom | M | 6 | 9 | Yes | Yes |  |  |  |  |
| *RASGRP1* | p.R34X | ST | Hemi | M | 3 | 89 | Yes |  |  |  | Yes |  |
| *RASGRP1* | p.214I | MS | Hemi | F | 42 | 72 |  |  |  |  |  |  |
| *RASGRP1* | p.T312A | MS | Hemi | M | 98 | 52 | Yes |  |  |  |  |  |
| *STK4* | c.1362+28055A>G | SP | Hom | M | 6 | 213 | Yes |  |  |  |  |  |
| *STK4* | c.1362+28055A>G | SP | Hom | F | 12 | 152 | Yes |  |  |  |  |  |
| *STK4* | c.360+5G>A | SP | Hom | M | 11 | 105 |  |  | Yes |  |  |  |
| *STK4* | p.L275Rfs20X | FS | Hom | F | 7 | 95 |  |  | Yes |  |  |  |
| *STK4* | p.L275Rfs20X | FS | Hom | M | 14 | 103 |  |  | Yes |  |  |  |
| *STK4* | p.M1L | MS | Hom | F | 48 | 180 | Yes |  |  |  |  |  |
| *STK4* | p.M1L | MS | Hom | F | 24 | 204 | Yes |  |  | Yes |  |  |
| *STK4* | p.Q274fs295X | FS | Hom | F | 5 | 25 |  |  | Yes |  |  |  |
| *STK4* | p.Q274fs295X | FS | Hom | F | 1 | 132 | Yes |  |  |  | Yes |  |
| *STK4* | p.R148W | MS | Hom | M | 1 | 24 | Yes |  | Yes | Yes | Yes |  |
| *STK4* | p.W250X | ST | Hom | F | 6 | 85 |  |  | Yes |  |  |  |
| *STK4* | p.W250X | ST | Hom | F | 4 | 83 |  |  | Yes |  |  |  |
| *STK4* | p.W250X | ST | Hom | M | 7 | 96 |  |  | Yes |  |  |  |
| *WAS* | c.1069+1G>A | FS | Hemi | M | 7 | 72 | Yes |  |  | Yes |  |  |
| *WAS* | c.1338+1G>A | SP | Hemi | M | 3 | 10 | Yes |  |  | Yes |  |  |
| *WAS* | c.1339-1G>T | SP | Hemi | M | 1 | 1 | Yes | Yes |  | Yes |  |  |
| *WAS* | c.1453+1G>C | SP | Hemi | M | 9 | 38 | Yes |  |  |  |  |  |
| *WAS* | c.1453+1G>C | SP | Hemi | M | 2 | 8 |  |  | Yes | Yes |  |  |
| *WAS* | c.1453+2T>C | SP | Hemi | M | 1 | 7 |  |  | Yes | Yes |  |  |
| *WAS* | c.181+2T>G | SP | Hemi | M | 11 | 72 | Yes |  | Yes | Yes |  |  |
| *WAS* | c.360+1delG | SP | Hemi | M | 2 | 2 |  |  |  | Yes |  |  |
| *WAS* | c.777+1G>A | SP | Hemi | M | 2 | 34 |  | Yes | Yes | Yes |  |  |
| *WAS* | c.777+1G>A | SP | Hemi | M | 9 | 32 |  |  | Yes |  |  |  |
| *WAS* | c.777+1G>A | SP | Hemi | M | 7 | 12 |  |  | Yes |  |  |  |
| *WAS* | c.777+1G>C | SP | Hemi | M | 5 | 9 |  |  |  | Yes |  |  |
| *WAS* | c.789+1G>C | SP | Hemi | M | 6 | 6 |  |  | Yes |  |  |  |
| *WAS* | EX11-12del | LD | Hemi | M | 2 | 21 |  |  | Yes |  |  |  |
| *WAS* | IVS10+1G>ASPLICING | SP | Hemi | M | 2 | 45 | Yes |  |  | Yes |  |  |
| *WAS* | IVS3-2A>G | SP | Hemi | M | 1 | 6 |  |  | Yes | Yes |  |  |
| *WAS* | IVS4-7T>G | SP | Hemi | M | 36 | 120 |  |  | Yes | Yes |  |  |
| *WAS* | IVS4-7T>G | SP | Hemi | M | 48 | 72 | Yes | Yes |  |  |  |  |
| *WAS* | IVS9+1G>C | SP | Hemi | M | 1 | 1 | Yes |  |  | Yes |  |  |
| *WAS* | IVS9+1G>C | SP | Hemi | M | 7 | 7 | Yes |  |  | Yes |  |  |
| *WAS* | p.A134T | MS | Hemi | M | 1 | 1 | Yes |  |  | Yes |  |  |
| *WAS* | p.A134T | MS | Hemi | M | 1 | 1 | Yes |  |  | Yes |  |  |
| *WAS* | p.A56V | MS | Hemi | M | 12 | 95 |  | Yes |  | Yes |  |  |
| *WAS* | p.E133K | MS | Hemi | M | 10 | 32 | Yes |  |  |  |  |  |
| *WAS* | p.E133K | MS | Hemi | M | 2 | 12 | Yes |  |  |  |  |  |
| *WAS* | p.E133K | MS | Hemi | M | 0.1 | 24 |  | Yes | Yes | Yes |  |  |
| *WAS* | p.E133K | MS | Hemi | M | 6 | 11 |  |  | Yes |  |  |  |
| *WAS* | p.E133K | MS | Hemi | M | 0.2 | 13 |  |  | Yes |  |  |  |
| *WAS* | p.E31K | MS | Hemi | M | 1 | 16 |  |  | Yes |  |  |  |
| *WAS* | p.E31K | MS | Hemi | M | 1 | 1 | Yes |  |  | Yes |  |  |
| *WAS* | p.E31K | MS | Hemi | M | 1 | 1 | Yes |  |  | Yes |  |  |
| *WAS* | p.E31K | MS | Hemi | M | 6 | 84 | Yes |  |  | Yes |  |  |
| *WAS* | p.E31K | MS | Hemi | M | 0.5 | 48 | Yes |  |  | Yes |  |  |
| *WAS* | p.F117S | MS | Hemi | M | 1.5 | 6 | Yes |  |  | Yes |  |  |
| *WAS* | p.G11RfsX27 | FS | Hemi | M | 1 | 5 | Yes |  |  | Yes |  |  |
| *WAS* | p.G11RfsX27 | FS | Hemi | M | 1 | 1 | Yes |  |  | Yes |  |  |
| *WAS* | p.G424AfsX20 | FS | Hemi | M | 9 | 30 |  |  | Yes |  |  |  |
| *WAS* | p.G432EfsX12 | FS | Hemi | M | 4 | 6 | Yes |  |  | Yes |  |  |
| *WAS* | p.G4AfsX41 | FS | Hemi | M | 7 | 12 | Yes |  | Yes | Yes |  |  |
| *WAS* | p.G4AfsX41 | FS | Hemi | M | 29 | 33 |  | Yes | Yes | Yes |  |  |
| *WAS* | p.G4AfsX41 | FS | Hemi | M | 1 | 1 |  | Yes |  | Yes |  |  |
| *WAS* | p.G60GlufsX16 | FS | Hemi | M | 4 | 5 |  |  | Yes | Yes |  |  |
| *WAS* | p.G60GlufsX16 | FS | Hemi | M | 5 | 48 | Yes |  |  |  | Yes |  |
| *WAS* | p.G70E | MS | Hemi | M | 1 | 3 | Yes |  |  | Yes |  |  |
| *WAS* | p.I294T | MS | Hemi | M | 8 | 24 |  |  | Yes |  |  |  |
| *WAS* | p.K230fsX262 | FS | Hemi | M | 9 | 37 | Yes |  |  |  |  |  |
| *WAS* | p.L215AfsX7 | FS | Hemi | M | 4 | 132 | Yes |  |  | Yes |  |  |
| *WAS* | p.M474T | MS | Hemi | M | 1 | 1 |  |  |  | Yes |  |  |
| *WAS* | p.M474T | MS | Hemi | M | 1 | 1 |  |  |  | Yes |  |  |
| *WAS* | p.M694V | MS | Hemi | M | 270 | 285 | Yes |  |  | Yes |  |  |
| *WAS* | p.M694V | MS | Hemi | M | 2 | 240 | Yes |  |  | Yes |  |  |
| *WAS* | p.M694V | MS | Hemi | M | 108 | 171 | Yes |  |  | Yes |  |  |
| *WAS* | p.M6NfsX32 | FS | Hemi | M | 1 | 110 | Yes |  |  | Yes |  |  |
| *WAS* | p.P354TfsX141 | FS | Hemi | M | 3 | 168 | Yes |  |  | Yes |  |  |
| *WAS* | p.P372LfsX73 | FS | Hemi | M | 1 | 4 | Yes |  |  | Yes |  |  |
| *WAS* | p.P413GfsX38 | FS | Hemi | M | 0 | 3 | Yes | Yes | Yes | Yes |  |  |
| *WAS* | p.P59LfsX17 | FS | Hemi | M | 0 | 10 | Yes | Yes | Yes | Yes |  |  |
| *WAS* | p.Q52_Y54delinsH | ID | Hemi | M | 2 | 3 | Yes |  | Yes | Yes |  |  |
| *WAS* | p.Q80X | ST | Hemi | M | 3 | 4 | Yes |  | Yes |  |  |  |
| *WAS* | p.Q91H | MS | Hemi | M | 1 | 16 | Yes |  |  | Yes |  |  |
| *WAS* | p.Q91H | MS | Hemi | M | 1 | 21 | Yes |  | Yes | Yes | Yes |  |
| *WAS* | p.Q91H | MS | Hemi | M | 89 | 90 |  |  | Yes |  |  |  |
| *WAS* | p.Q91X | ST | Hemi | M | 1 | 3 | Yes |  |  | Yes |  |  |
| *WAS* | p.Q91X | ST | Hemi | M | 1 | 16 | Yes |  | Yes | Yes |  |  |
| *WAS* | p.Q91X | ST | Hemi | M | 1 | 2 |  | Yes | Yes | Yes |  |  |
| *WAS* | p.Q91X | ST | Hemi | M | 1 | 3 | Yes | Yes |  | Yes |  |  |
| *WAS* | p.Q91X | ST | Hemi | M | 2 | 3 | Yes |  |  | Yes |  |  |
| *WAS* | p.R138P | MS | Hemi | M | 3.5 | 26 | Yes |  |  | Yes |  |  |
| *WAS* | p.R13X | ST | Hemi | M | 1 | 2 | Yes |  | Yes | Yes |  |  |
| *WAS* | p.R211X | ST | Hemi | M | 3 | 3 |  |  | Yes | Yes |  |  |
| *WAS* | p.R211X | ST | Hemi | M | 1 | 15 |  |  | Yes | Yes |  |  |
| *WAS* | p.R304P | MS | Hemi | M | 0.5 | 2 | Yes |  |  | Yes |  |  |
| *WAS* | p.R321X | ST | Hemi | M | 2 | 84 | Yes |  | Yes | Yes |  |  |
| *WAS* | p.R321X | ST | Hemi | M | 1 | 7 |  |  | Yes |  |  |  |
| *WAS* | p.R321X | ST | Hemi | M | 2 | 32 |  |  | Yes |  |  |  |
| *WAS* | p.R34X | ST | Hemi | M | 1 | 3 | Yes |  |  | Yes |  |  |
| *WAS* | p.R34X | ST | Hemi | M | 1 | 1 |  |  |  | Yes |  |  |
| *WAS* | p.R86H | MS | Hemi | M | 7 | 72 |  |  |  | Yes |  |  |
| *WAS* | p.R86H | MS | Hemi | M | 1 | 2 | Yes |  |  | Yes |  |  |
| *WAS* | p.R86H | MS | Hemi | M | 1 | 12 |  | Yes | Yes | Yes |  |  |
| *WAS* | p.R86H | MS | Hemi | M | 1 | 3 | Yes |  |  | Yes |  |  |
| *WAS* | p.T45M | MS | Hemi | M | 6 | 108 | Yes |  |  | Yes |  |  |
| *WAS* | p.T45M | MS | Hemi | M | 6 | 48 | Yes |  |  | Yes |  |  |
| *WAS* | p.T45M | MS | Hemi | M | 9 | 36 |  |  |  | Yes |  |  |
| *WAS* | p.T45M | MS | Hemi | M | 6 | 132 |  |  |  | Yes |  |  |
| *WAS* | p.T481 | MS | Hemi | M | 1 | 1 | Yes |  |  | Yes |  |  |
| *WAS* | p.T481 | MS | Hemi | M | 1.5 | 84 | Yes |  |  | Yes |  |  |
| *WAS* | p.T481 | MS | Hemi | M | 8 | 24 | Yes |  |  | Yes |  |  |
| *WAS* | p.T481 | MS | Hemi | M | 1 | 60 | Yes |  |  | Yes |  |  |
| *WAS* | p.T481 | MS | Hemi | M | 1.5 | 48 | Yes |  |  | Yes |  |  |
| *WAS* | p.T481 | MS | Hemi | M | 2 | 24 | Yes |  |  | Yes |  |  |
| *WAS* | p.V141E | MS | Hemi | M | 0 | 6 |  |  | Yes | Yes |  |  |
| *WAS* | p.V247WfsX15 | FS | Hemi | M | 10 | 60 | Yes |  |  | Yes |  |  |
| *WAS* | p.V247WfsX15 | FS | Hemi | M | 9 | 30 |  |  | Yes |  |  |  |
| *WAS* | p.V332A | MS | Hemi | M | 20 | 38 | Yes |  |  | Yes |  |  |
| *WAS* | p.VZSM | MS | Hemi | M | 6 | 117 | Yes |  |  |  |  |  |
| *WAS* | p.Y83S | MS | Hemi | M | 8 | 29 |  |  | Yes | Yes |  |  |
| *WDR1* | p.D572V | MS | Hom | F | 54 | 84 | Yes |  |  |  |  |  |
| *WIPF1* | p.S434X | ST | Hom | F | 96 | 144 | Yes |  |  |  |  |  |
| *WIPF1* | p.R158X | ST | Hom | M | 30 | 132 | Yes | Yes |  | Yes |  |  |
| *WIPF1* | p.R438X | ST | Hom | F | 3 | 105 | Yes |  |  | Yes |  |  |
| *WIPF1* | p.S350 | ST | Hom | M | 4 | 36 | Yes | Yes |  | Yes |  |  |
| SP: splicing mutation, FS: Frame-shift mutation, MS: Missense mutation, LD: large deletion, ST: Stop-gain mutations, ID: in-frame deletion, Hom: Homozygous, Hemi: Hemizygous, Het: Heterozygous, Comp het: Compound heterozygous, M: Male, F: Female. | | | | | | | | | | | | |

**
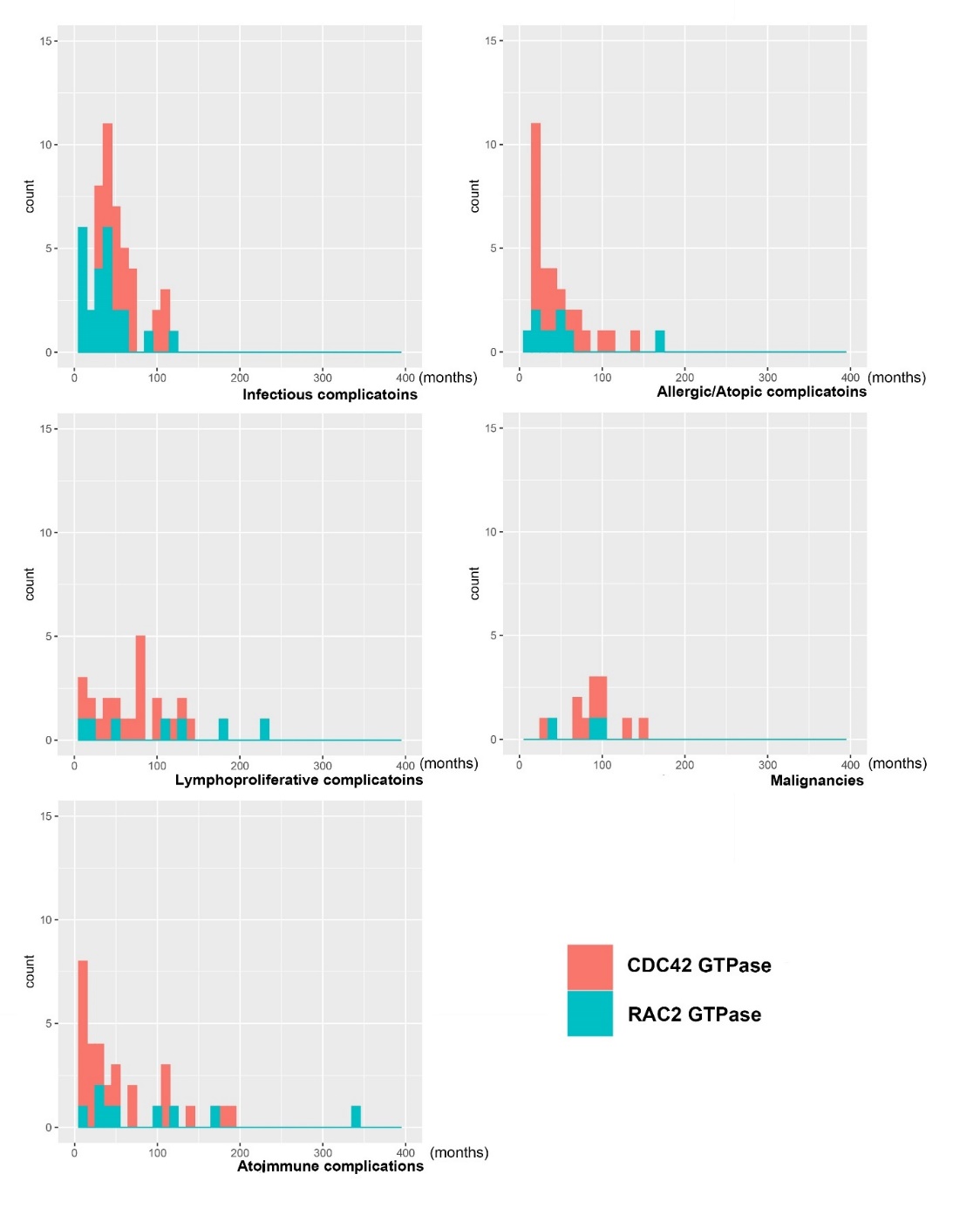
**

**Figure S1-** Longitudinal infectious and non-infectious clinical manifestations of 2 main subcategories of patients with monogenic actin-related inborn errors of immunity.

**
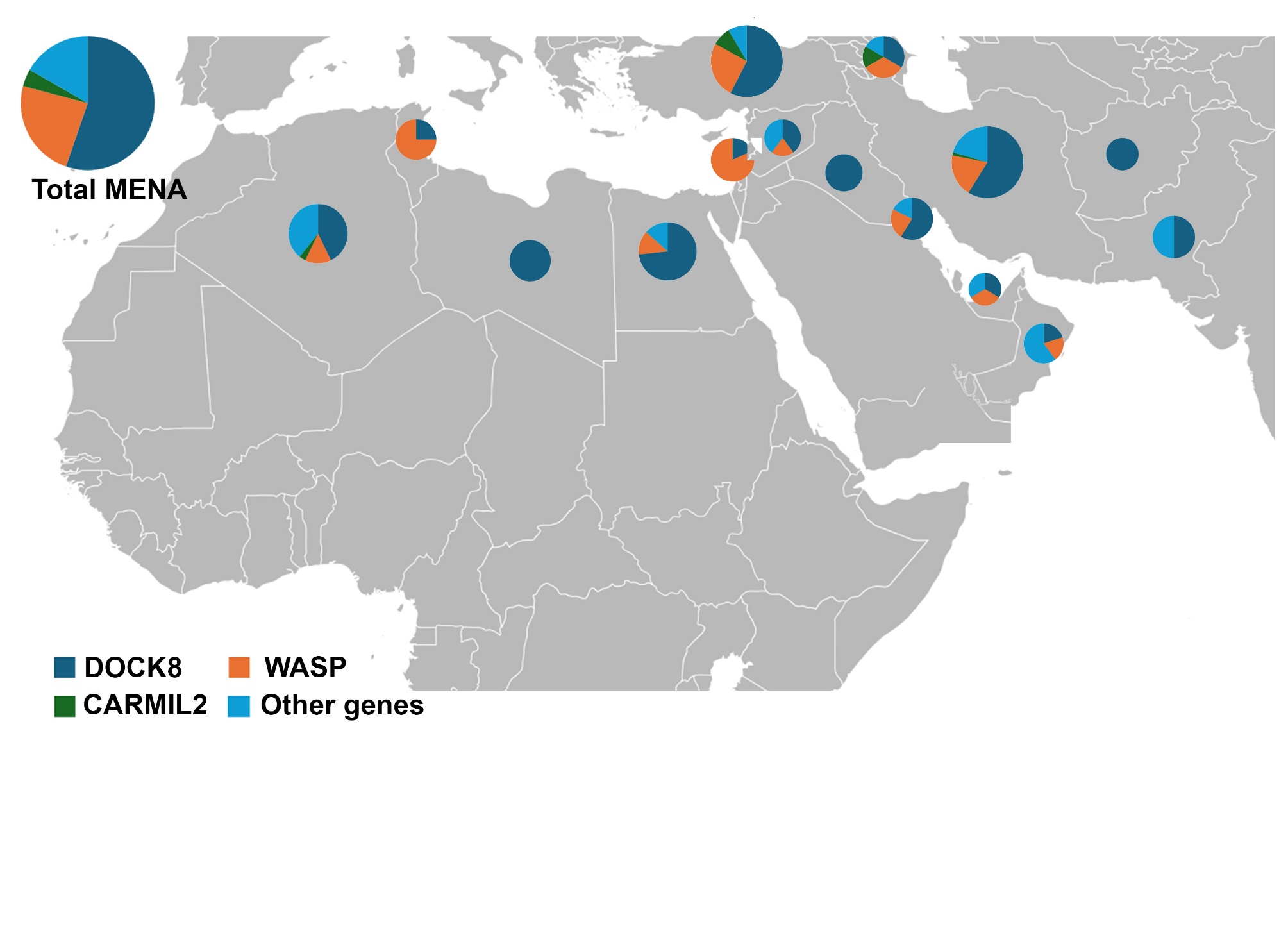
**

**Figure S2-** Pie charts depict the proportion of DOCK8, WASP and CARMIL2 deficiencies and other rare genetic defects in different population level of the countries studied in comparison with total MENA region.

**
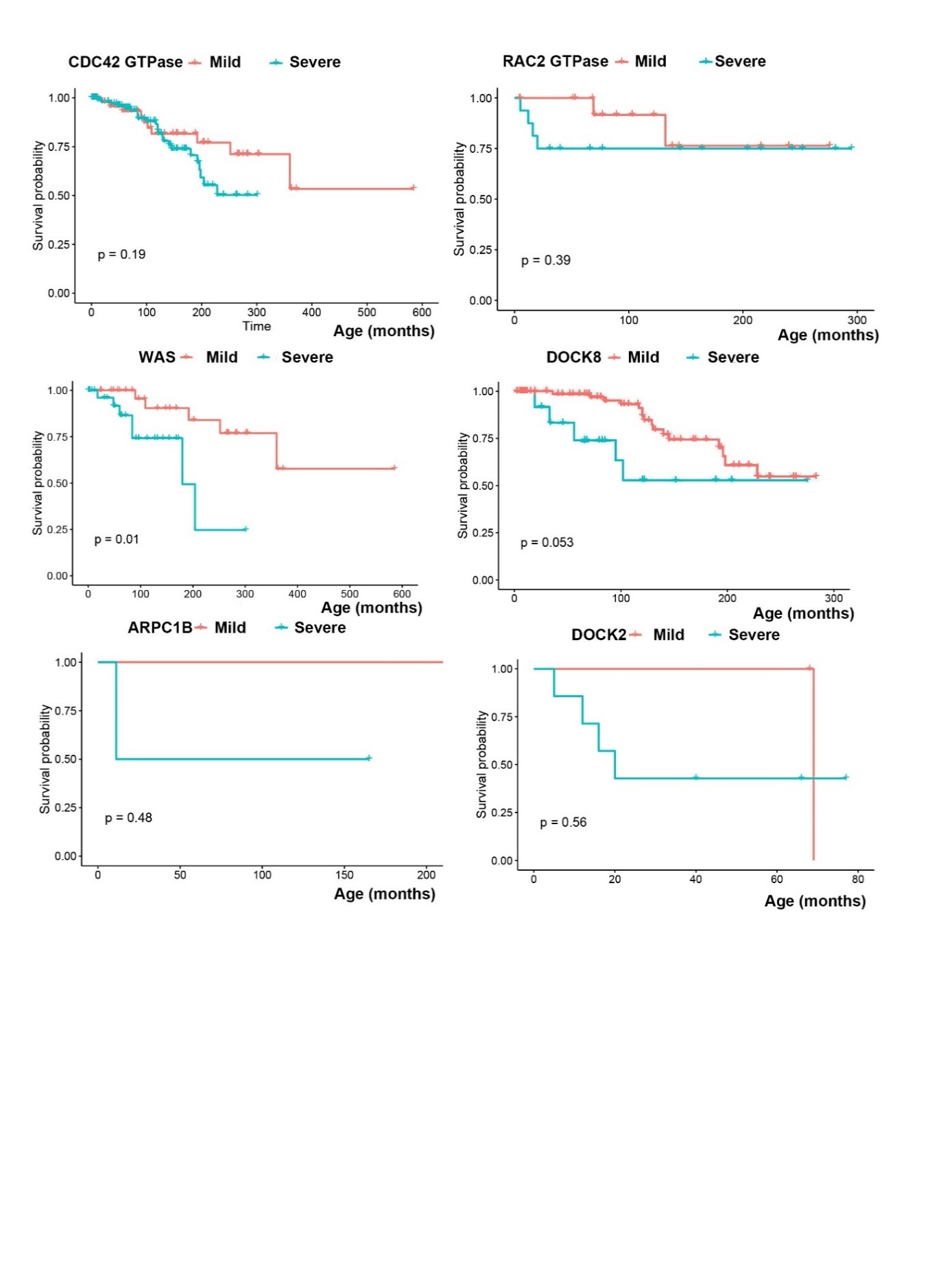
**

**Figure S3-** Survival analysis of monogenic actin-related inborn errors of immunity in the MENA region based on the severity of mutations observed in the main two groups of GTPase (CDC42 and RAC2) and frequent genetic defects (*WAS, DOCK8, ARPC1B* and *DOCK2*).

**Figure S4-** Survival analysis of monogenic actin-related inborn errors of immunity in the MENA region based on the molecular diagnosis classification in three groups of GTPase CDC42, GTPase RAC2 and transcription/elongation of actin cytoskeleton.

**
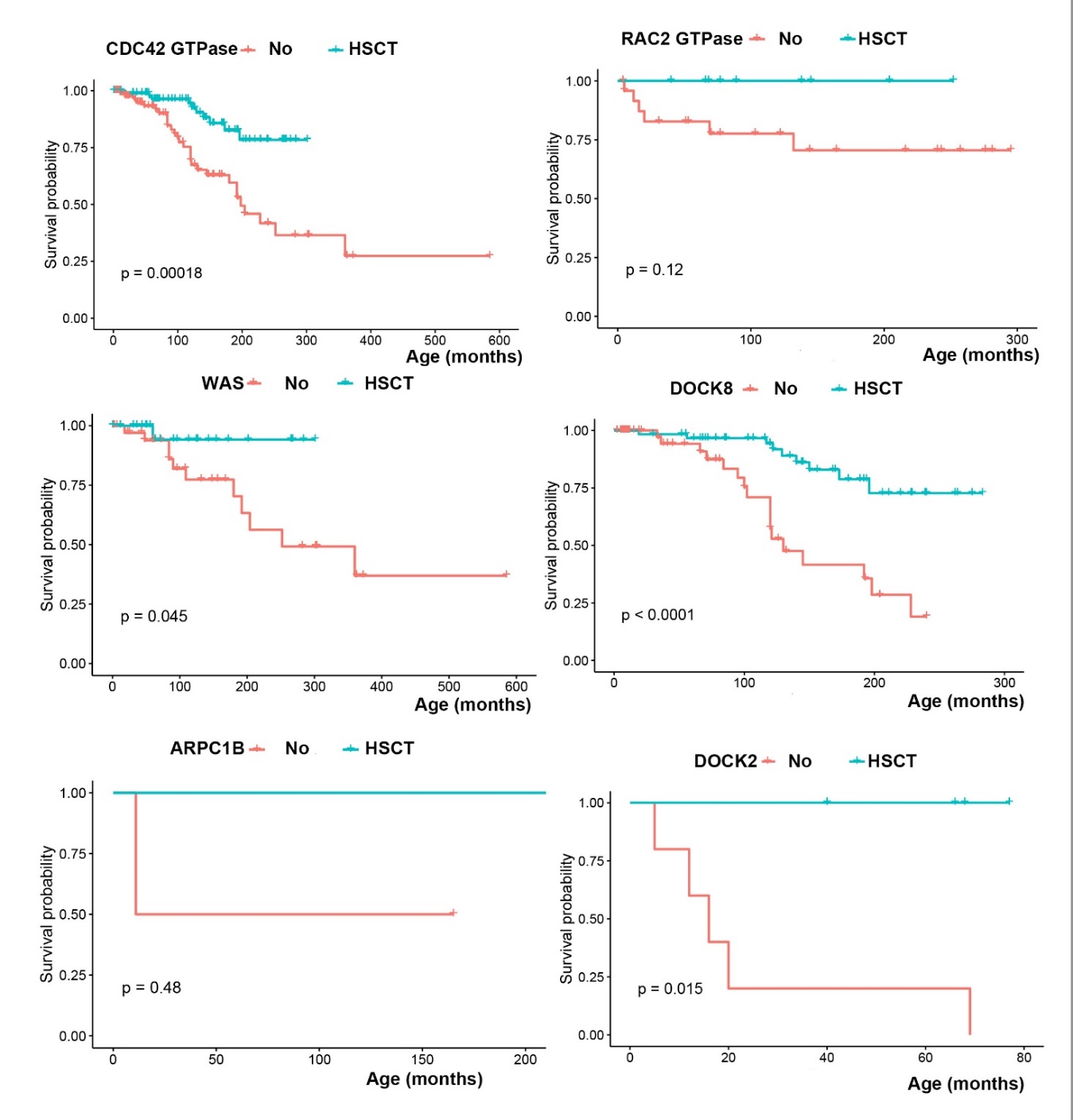
**

**Figure S5-** Survival analysis of actin-related inborn errors of immunity in the MENA region based on the HSCT outcome observed in main two groups of GTPase (CDC42 and RAC2) and frequent genetic defects (*WAS, DOCK8, ARPC1B* and *DOCK2*).
